# Optimization of *Agrobacterium*-Mediated Transformation in Soybean

**DOI:** 10.3389/fpls.2017.00246

**Published:** 2017-02-24

**Authors:** Shuxuan Li, Yahui Cong, Yaping Liu, Tingting Wang, Qin Shuai, Nana Chen, Junyi Gai, Yan Li

**Affiliations:** National Key Laboratory of Crop Genetics and Germplasm Enhancement, Key Laboratory for Biology and Genetic Improvement of Soybean (General, Ministry of Agriculture), National Center for Soybean Improvement, Jiangsu Collaborative Innovation Center for Modern Crop Production, Nanjing Agricultural UniversityNanjing, China

**Keywords:** *Agrobacterium*, efficiency, half-seed explants, infection, regeneration, shoot elongation, soybean transformation

## Abstract

High transformation efficiency is a prerequisite for study of gene function and molecular breeding. *Agrobacterium tumefaciens*-mediated transformation is a preferred method in many plants. However, the transformation efficiency in soybean is still low. The objective of this study is to optimize *Agrobacterium*-mediated transformation in soybean by improving the infection efficiency of *Agrobacterium* and regeneration efficiency of explants. Firstly, four factors affecting *Agrobacterium* infection efficiency were investigated by estimation of the rate of GUS transient expression in soybean cotyledonary explants, including *Agrobacterium* concentrations, soybean explants, *Agrobacterium* suspension medium, and co-cultivation time. The results showed that an infection efficiency of over 96% was achieved by collecting the *Agrobacterium* at a concentration of OD_650_ = 0.6, then using an *Agrobacterium* suspension medium containing 154.2 mg/L dithiothreitol to infect the half-seed cotyledonary explants (from mature seeds imbibed for 1 day), and co-cultured them for 5 days. The *Agrobacterium* infection efficiencies for soybean varieties Jack Purple and Tianlong 1 were higher than the other six varieties. Secondly, the rates of shoot elongation were compared among six different concentration combinations of gibberellic acid (GA_3_) and indole-3-acetic acid (IAA). The shoot elongation rate of 34 and 26% was achieved when using the combination of 1.0 mg/L GA_3_ and 0.1 mg/L IAA for Jack Purple and Tianlong 1, respectively. This rate was higher than the other five concentration combinations of GA_3_ and IAA, with an 18 and 11% increase over the original laboratory protocol (a combination of 0.5 mg/L GA_3_ and 0.1 mg/L IAA), respectively. The transformation efficiency was 7 and 10% for Jack Purple and Tianlong 1 at this optimized hormone concentration combination, respectively, which was 2 and 6% higher than the original protocol, respectively. Finally, GUS histochemical staining, PCR, herbicide (glufosinate) painting, and QuickStix Kit for Liberty Link (*bar*) were used to verify the positive transgenic plants, and absolute quantification PCR confirmed the exogenous gene existed as one to three copies in the soybean genome. This study provides an improved protocol for *Agrobacterium*-mediated transformation in soybean and a useful reference to improve the transformation efficiency in other plant species.

## Introduction

Soybean [Glycine max (L.)] is one of the most important oil crops and a significant source of protein for food and feed in the world. Soybean seeds are not only rich in essential amino acids, but also rich in dietary minerals, vitamins, unsaturated fatty acids, and isoflavones, which are implicated as beneficial food for human health (Han et al., 2003). Genetically modified (GM) soybean is one of the earliest introduced GM crops for commercial cultivation and the largest GM crop in terms of acreage planted worldwide (Yang et al., 2012). Studies have shown that the new GM crop varieties with important application values must be selected from hundreds, thousands, or even tens of thousands transformation events (Wang et al., 2006). On the other hand, although the soybean genome sequence has been released (Schmutz et al., 2010), the functions of most soybean genes are unknown. Therefore, an efficient and stable genetic transformation method is an important prerequisite to study gene functions in soybean and develop new soybean varieties by molecular breeding.

There are three main ways to deliver foreign DNA into host plants, including *Agrobacterium*-mediated transformation (Hinchee et al., 1988), particle bombardment (Mccabe et al., 1988), and pollen-tube pathway method (Shou et al., 2002). *Agrobacterium tumefaciens* is a gram-negative bacterium and widespread in soil. *Agrobacterium* can infect plants through its Ti plasmid, and its T-DNA can be integrated to the host plant genome and inherited by the offspring of host plant (Wu et al., 2005). Particle bombardment method can insert the exogenous DNA (which is attached to micron-sized metal particles) directly into the tissue cells using high-pressure helium gas (Liu et al., 2001). Due to the strong penetrating power of small particle, the exogenous DNA-metal particle can penetrate the cell wall and cell membrane into cells. Particle bombardment transformation makes the transfer of DNA is no longer restricted by species or genotypes, especially suitable for the species and genotypes which are not sensitive to *Agrobacterium* infection (Wang et al., 2002). However, the cost of particle bombardment transformation is high, and gene rearrangement and high copy numbers are often observed in transgenic plants using this method (Wang et al., 2009). Pollen-tube pathway method delivers the exogenous DNA into a zygote cell or early embryo cells of recipient plant through the pollen tube directly (Zhou et al., 1983), which does not need tissue culture process (Ren et al., 2012), and exogenous DNA can be transferred to host plants directly without vector construct (Xiao et al., 2007). But the exogenous gene needs to go through many barriers along pollen tube into the zygote, and can be destroyed by the nuclease in stigma. Therefore, it is difficult to get a stable transformation rate by this method (Shou et al., 2002; Dong et al., 2011).

About 85% of the transgenic plants are obtained using the *Agrobacterium*-mediated transformation method (Yu et al., 2010). *Agrobacterium*-mediated transformation is the best choice for plant transformation due to its simple operation, high reproducibility, low copy number, and low experimental cost. This method also can transfer a large fragment of foreign gene into the host plant genome. *Agrobacterium*-mediated transformation is also a preferred method in soybean. Hinchee et al. used soybean cotyledonary nodes as the explants to obtain transgenic soybean plants (Hinchee et al., 1988). From then on, many researchers used cotyledonary nodes as the explants for transformation (Di et al., 1996; Zhang et al., 1999). In addition to cotyledonary nodes, many other tissue types can be used as explants, such as primary leaf nodes, epicotyls, and hypocotyls, immature embryos, axillary buds, and stem tips. But the regeneration efficiency differed greatly among different explants. Cotyledonary nodes have a higher regeneration efficiency than other types of explants in soybean (Kim et al., 1990; Sato et al., 1993; Liu et al., 2004; Zhong and Que, 2009). In addition, using cotyledonary nodes as the explants has several other advantages. First, it is easy to obtain the cotyledonary nodes by germinating soybean seeds, which is not limited by the season. Second, the regeneration process is simple, including shoot induction, elongation and root induction. However, the transformation rate of this method is too low. Instead of germinating the soybean seeds to get cotyledonary nodes, Paz et al. imbibed mature soybean seeds for about 24 h to obtain the “half seeds” as the cotyledonary explants (Paz et al., 2006). The overall average transformation efficiency was 3.8% using this method, which was 1.5 times higher than using the cotyledonary nodes from the 5- to 7-d-old seedlings (Paz et al., 2006), but still low compared with the transformation efficiency in other crops such as rice 23% (Lin and Zhang, 2005; Ge et al., 2006) and maize 30–40% (Ishida et al., 1996; Yang et al., 2006). The previous study indicates that the low shoot elongation rate during explants regeneration is a bottleneck for soybean transformation (Song et al., 2013).

**Figure 1:**
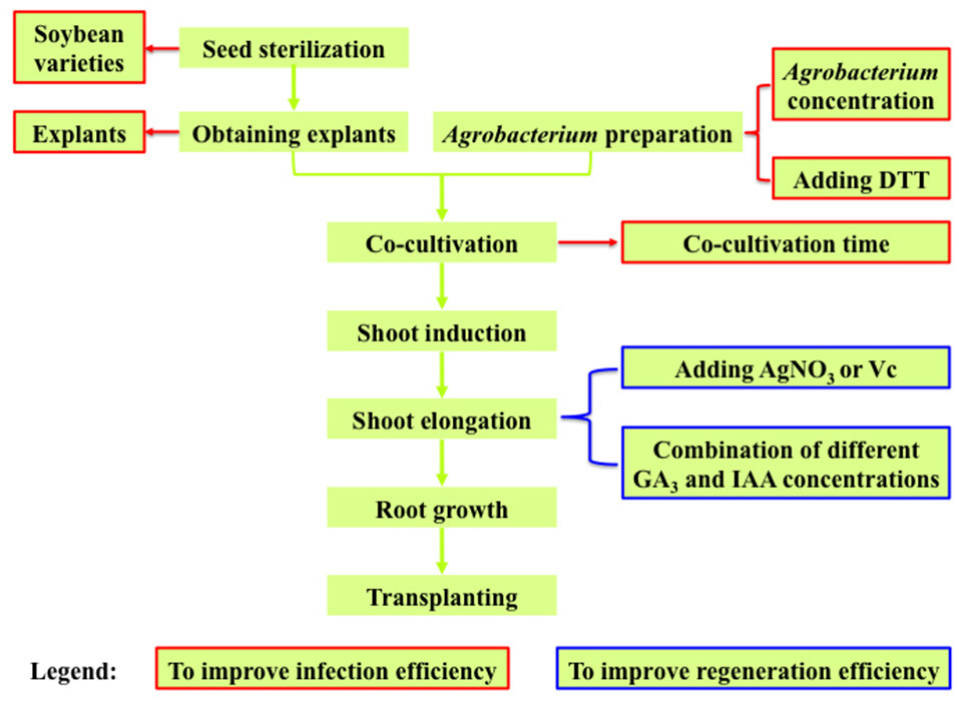
**Experimental design for optimizing ***Agrobacterium***-mediated soybean transformation in this study**.

During the transformation process, there are many factors affecting the efficiency. The general transformation process includes the following steps: obtain the explants after seed sterilization; infect explants with the *Agrobacterium* suspension liquid and co-cultivate them on the co-cultivation medium (CCM) with their adaxial side (flat side) upwards (Gao et al., 2015); transfer the *Agrobacterium*-infected explants to shoot induction medium (SIM); transfer the explants to shoot elongation medium (SEM); put the elongated shoots into rooting medium; eventually the plants were transferred to pots and grown to maturity (Figure 1). During this process, many factors such as type of explants, concentration of *Agrobacterium* for infection, co-cultivation time, and medium composition (including CCM, SIM, SEM, and rooting medium), will affect the transformation efficiency. The efficiencies of regeneration and transformation also varied among different soybean genotypes (Hinchee et al., 1988; Bailey and Parrott, 1993; Donaldson and Simmonds, 2000; Yang et al., 2016). The overall transformation efficiency depends on the efficiencies of *Agrobacterium* infection and explant regeneration.

*Agrobacterium* concentration is an important factor affecting its infection efficiency (Paz et al., 2006). *Agrobacterium* concentration reflects its growth status and the *Agrobacterium* during logarithmic growth phase is thought to have higher infection ability (Zhou et al., 2011). In the *Vanda Kasem's Delight Orchid*, the highest β-glucuronidase (GUS) expression in protocorm-like bodies was observed when the optical density at 600 nm (OD_600_) of *Agrobacterium* suspension was 0.8 (Gnasekaran et al., 2014). In groundnut, the most suitable OD_600_ of *Agrobacterium* for infection was determined as 1.8 (Tiwari et al., 2015). The *Agrobacterium* concentration for infection of ramie was optimized as OD_600_ = 0.6 (An et al., 2014).

Plant hormones play important roles in explant regeneration during tissue culture, especially auxins, cytokinins, and gibberellins. Auxin was discovered as a phytohormone with the chemical structure of indole-3-acetic acid (IAA), and synthetic auxins such as indole-3-butyric acid (IBA), naphthalene acetic acid (NAA), and 2,4-dichlorophenoxyacetic acid (2,4-D) also have auxin activity, which promote cell elongation, plant growth, and development (Normanly, 1997). Cytokinins promote cytokinesis, differentiation and growth of various tissues (Letham, 1967). 6-benzylaminopurine (6-BA), kinetin (KT), zeatin (ZT) are synthetic cytokinins. In plant tissue culture, the concentrations of auxin and cytokinin affect the regeneration efficiency of explants (Skoog and Miller, 1957). Kumari et al. found that Murashige and Skoog (MS) medium (Murashige and Skoog, 1962) supplemented with 2 mg/L 6-BA and 0.2 mg/L IAA was optimum for shoot regeneration in *Bacopa monniera* (Kumari et al., 2015). In groundnut, a high regeneration efficiency was achieved by adding 66.6 μM 6-BA in the medium, while the highest number of shoot buds per explant was achieved by adding 20 μM 6-BA and 10 μM 2,4-D (Tiwari et al., 2015). The highest regeneration efficiency for cotyledonary nodes of Crambe was observed on basic medium with 0.5 μM NAA and 2.2 μM 6-BA (Qi et al., 2014).

Gibberellin (GA) is one of the most important hormones affecting plant growth and development (Hua and Irving, 2011). It not only promotes seed germination, hypocotyl elongation, xylem development, and internode elongation, but also induces the differentiation of flower buds (Yukika et al., 1997; Almqvist, 2003; Thomas and Sun, 2004). GA is necessary for stem elongation (Nishijima et al., 1997). A previous study showed that the highest number of shoots per explant and shoot elongation rate was obtained by using 3 mg/L 6-BA in combination with 0.5 mg/L GA_3_ during shoot induction stage in tea (Gonbad et al., 2014). MS medium supplemented with 1 mg/L 6-BA, 0.1 mg/L IBA, and 2 mg/L GA_3_ promoted shoot elongation significantly in *Cerasus campanulata* (Wang and Huang, 2002). In sweet potato, the combination of 10 mg/L GA_3_ and 1 mg/L 6-BA could give rise to significantly taller shoots (20 mm) compared to the rest of the treatments (Masekesa et al., 2016).

Callus browning is another problem to solve for *Agrobacterium*-mediated transformation. Explants will have defense response when infected by *Agrobacterium*. Then the protective layer is formed on the cell surface that results in browning or necrosis. In order to mitigate tissue browning in *Agrobacterium*-mediated transformation, researchers have concentrated on the antioxidants such as ascorbate (Vc), α-lipoic acid, α-tocopherol, dithiothreitol (DTT), glutathione, L-cysteine (L-cys), polyvinylpolypyrrolidone (PVPP), and selenite (Dan, 2008). These antioxidants can reduce tissue browning of transformed cells and improve regeneration efficiency, thus enhancing transformation efficiency. A combination of Vc, silver nitrate (AgNO_3_) and cysteine improved *Agrobacterium*-mediated transformation efficiency in sugar cane (Enríquez Obregón et al., 1998). Adding Vc to tissue culture medium can significantly reduce the degree of browning, and adding AgNO_3_ can not only reduce tissue browning effectively, but also improve the number of adventitious buds significantly in *Zizyphus jujube* (Huang et al., 2006). When AgNO_3_ and 6-BA were added in the culture medium during soybean transformation using hypocotyl as explants, the induction rate of adventitious buds was improved (five times higher than the control group) and the rate of hypocotyl browning was reduced (Wang and Xu, 2008). Adding α-lipoic acid significantly increased the induction rate of transgenic shoots in four crop species including soybean, wheat, tomato, and cotton (Dan et al., 2009). DTT has been found to play positive roles in plant transformation. In soybean, different concentrations of DTT and L-cys were added in the *Agrobacterium* re-suspension medium and CCM to inhibit the necrosis of explants (Olhoft and Somers, 2001; Li et al., 2008b; Zhang et al., 2016). When 0.1% DTT was added in CCM, the regeneration efficiency increased from 8 to 22% in wheat (Yu et al., 2005).

In this study, we tried to improve the *Agrobacterium*-mediated transformation efficiency in soybean by improving the infection efficiency of *Agrobacterium* and regeneration efficiency of explants (shoot elongation rate). The *Agrobacterium* infection efficiencies were compared using different concentrations of *Agrobacterium* for infection, explants, *Agrobacterium* suspension medium, co-cultivation time, and soybean varieties. The rates of shoot elongation were evaluated by adding different concentration combinations of GA_3_ and IAA, as well as AgNO_3_ or Vc in the tissue culture medium. The *Agrobacterium* infection efficiency was estimated by the rate of GUS transient expression in soybean cotyledonary explants, and the rate of shoot elongation was investigated by the frequencies of elongated shoots. Finally, the transformation efficiency was calculated based on the number of positive transgenic soybean seedlings detected by different methods. The optimized protocol for *Agrobacterium*-mediated transformation in this study would be helpful to further study gene functions in soybean and develop new varieties by molecular breeding.

## Materials and methods

### Experimental design

As the overall transformation efficiency is largely determined by the efficiencies of *Agrobacterium* infection and explant regeneration (where shoot elongation rate is the bottleneck in soybean), we investigated the key factors affecting *Agrobacterium* infection efficiency including *Agrobacterium* concentrations, explants, *Agrobacterium* suspension medium, co-cultivation time, and soybean varieties (Figure 1, textboxes with red borders), and the major factors affecting the explant regeneration efficiency (shoot elongation rate) including different concentration combinations of GA_3_ and IAA in SEM as well as adding AgNO_3_ or Vc in SIM and SEM (Figure 1, textboxes with blue borders).

### Plant materials

Soybean varieties including Tianlong 1, Jack Purple, DLH, NN419, Williams 82, HZM, NN34, NN88-1 were used in this study. The seeds were provided by the National Center for Soybean Improvement at Nanjing Agricultural University, Nanjing, China.

### *Agrobacterium* strain and vector

The *A. tumefaciens* strain EHA101 (Hood et al., 1986) and the binary plasmid pTF102 (Frame and Kan, 2002) were used in this study, which were kindly provided by Dr. Huixia Shou (Zhejiang University, China) and Dr. Kan Wang (Iowa State University, USA), respectively. The pTF102 vector contains a *GUS* gene (with intron) as the reporter gene, a *bar* gene as the selectable marker gene (conferring resistance to herbicide glufosinate), and an *aadA* gene for resistance to antibiotics spectinomycin.

### *Agrobacterium* culture and infection medium

To prepare the *Agrobacterium* infection medium, a single colony of *A. tumefaciens* strain EHA101/pTF102 was picked from the plate and put into 5 ml liquid YEB (An et al., 1989, 5 g/L NaCl, 10 g/L peptone, 5 g/L yeast extract) containing 25 mg/L chloramphenicol, 50 mg/L kanamycin, 50 mg/L streptomycin, and 100 mg/L spectinomycin, and cultured for 24 h at 28°C (250 rpm) to get the starter culture. Next, 200 ul of the starter culture was transferred to 200 ml YEB culture and grown at 28°C (250 rpm) in a shaker incubator until OD_650_ reached 0.6, 0.8, or 1.0. The *Agrobacterium* culture was collected and centrifuged at 5000 rpm (22°C) for 10 min, and then the pellet was re-suspended using the liquid CCM until OD_650_ reached 0.7. The re-suspended infection medium was shaken for 0.5 h (70 rpm, 22°C) before use. The liquid CCM contains 1/10X B_5_ basal medium (Gamborg and Al, 1968) supplemented with 3.9 g/L 2-[*N*-morpholino] ethanesulfonic acid (MES), 3% (30 g/L) sucrose, pH = 5.4 (adjusting by KOH), 1.67 mg/L 6-BA, 0.25 mg/L GA_3_, 40 mg/L acetosyringone (AS), and DTT (0 or 154.2 mg/L).

### The process of soybean transformation

**Figure 2:**
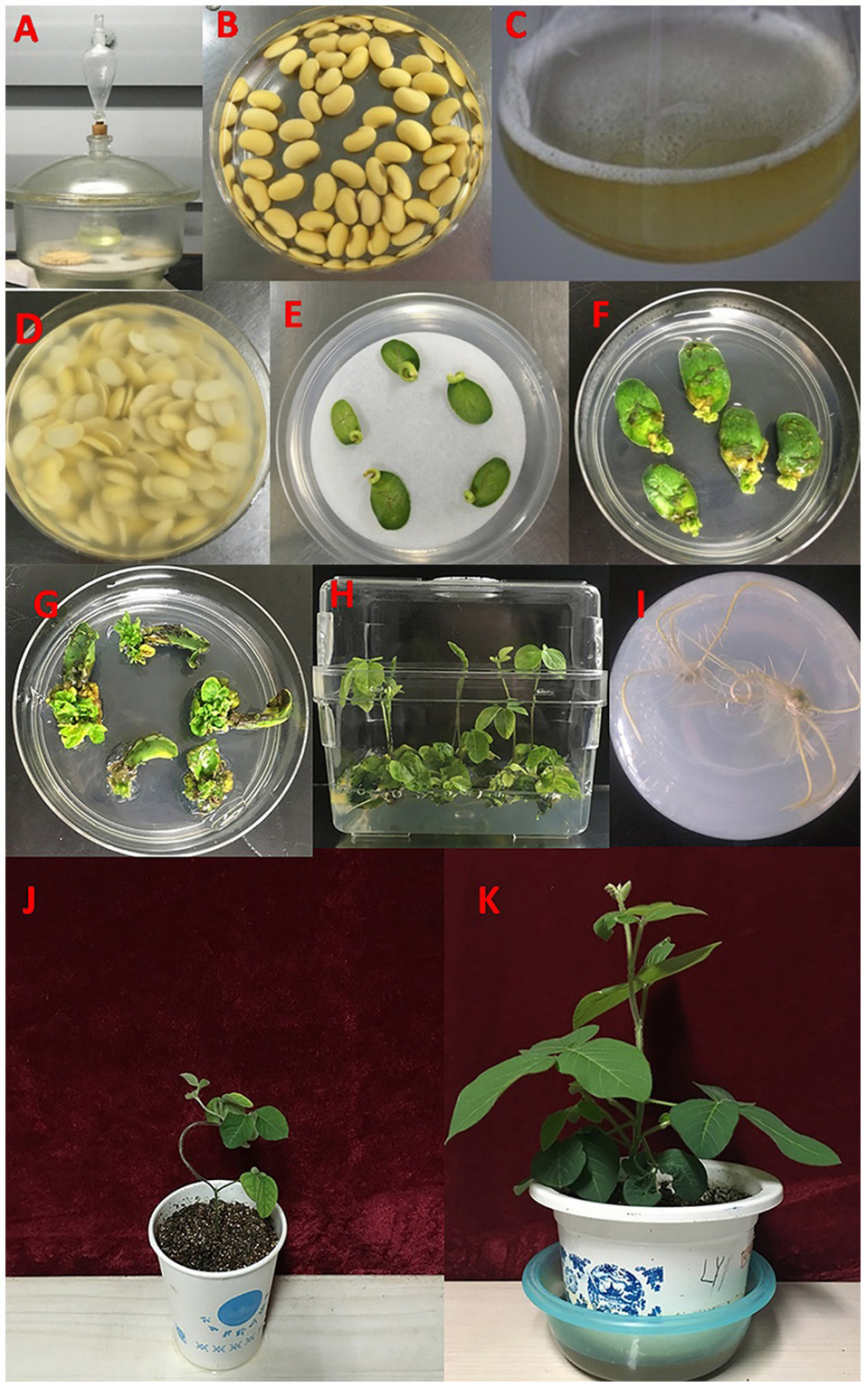
**The experimental process of ***Agrobacterium***-mediated transformation using half-seed soybean explants. (A)** Seed sterilization. **(B)** Seed imbibition. **(C)** Preparation of *Agrobacterium*. **(D)** *Agrobacterium* infection of half-seed cotyledonary explants. **(E)** Co-cultivation. **(F,G)** Shoot induction. **(H)** Shoot elongation. **(I)** Rooting. **(J,K)** Transplanting and adaptation to normal growth condition.

The protocol for *Agrobacterium*-mediated soybean transformation in this study was based on a previous study (Paz et al., 2006) with modification, which is briefly summarized as the following steps (Figure 2).

#### Explant preparation, *Agrobacterium* infection and co-cultivation

The mature soybean seeds without any defects were wiped clean using a cotton cloth (wetted by 75% alcohol), and then surface-sterilized for 6 or 12 h with chlorine gas (100 ml NaClO + 10 ml HCl or 100 ml NaClO + 3.5 ml HCl) in a tightly sealed chamber (Figure 2A). To get the half-seed cotyledonary explants, the sterilized seeds were soaked in sterile water at 23°C under dark for 16–18 h (Figure 2B). Then the imbibed seeds were longitudinally cut along the hilum using a scalpel, and the seed coats were removed. The hypocotyls were trimmed to 3 mm. The half-seed cotyledonary explants or cotyledonary nodes from 1-, 3-, or 5-d-old seedlings were put in the prepared re-suspended *Agrobacterium* infection medium (Figure 2C) for 30 min (Figure 2D). The *Agrobacterium* suspension medium was shaken during the period of infection to make the explants in well contact with the *Agrobacterium* liquid. After infection, five to seven explants (adaxial side up) were evenly placed on sterile filter paper over solid CCM in Petri dishes (90 mm in diameter × 15 mm deep), and co-cultivated at 23°C under a photoperiod of 16 h/8 h (light/dark) for 3–5 d (Figure 2E). The solid CCM contains 1/10X B_5_ basal medium supplemented with 3.9 g/L MES, 3% (30 g/L) sucrose, 0.5% (5 g/L) agrose (Biowest, Spain), pH = 5.4 (adjusting by KOH), 1.67 mg/L 6-BA, 0.25 mg/L GA_3_, 40 mg/L AS, 400 mg/L L-cys, 154.2 mg/L DTT, and 158 mg/L sodium thiosulfate.

#### Tissue culture and transplanting

After co-cultivation, the explants were inserted (tilting 45 degrees) in SIM with the adaxial side facing upward, and maintained in a walk-in chamber under the photoperiod of 16 h/8 h (light/dark) and temperature of 23°C (Figures 2F,G). The SIM contains B_5_ basal medium supplemented with 3% sucrose, 0.35% (3.5 g/L) phytagel (Sigma, USA), 0.58 g/L MES, pH 5.6, 1.67 mg/L 6-BA, 50 mg/L cefotaxime (Cef), 500 mg/L carbenicillin (Carb), and 5 mg/L glufosinate (Sigma, USA). Two weeks later, the explants were transferred to fresh SIM. Four weeks after shoot induction, the explants were transferred to SEM, which contains MS basal medium (Murashige and Skoog, 1962) supplemented with 3% sucrose, 0.35% (3.5 g/L) phytagel (Sigma, USA), 0.58 g/L MES, pH 5.6, 0.5 mg/L GA_3_, 0.1 mg/L IAA, 1 mg/L zeatin (ZR), 50 mg/L asparagine (Asp), 100 mg/L L-pyroglutamic acid (L-pyro), 75 mg/L Cef, 500 mg/L Carb, and 3 mg/L glufosinate. The explants were transferred to fresh SEM every two weeks until the elongated shoots reached 3 cm high (Figure 2H). Elongated shoots were placed into rooting medium (B_5_ basal medium supplemented with 1.5% sucrose, 0.8% agar powder, 0.59 g/L MES, pH 5.7, and 1 mg/L IBA), until the roots were developed to 2–3 cm in length (Figure 2I). Eventually the plants were transplanted in pots (soil: vermiculite = 1:1) and grown in greenhouse at 28/24°C with a photoperiod of 16 h/8 h (light/dark) until maturity (Figures 2J,K).

### Identification of positive transgenic soybean plants

We used the following four methods to detect the positive transgenic soybean plants.

#### GUS histochemical staining

The young leaves of transgenic soybean plants were collected and submerged in GUS staining solution (Table S1) overnight in dark at 37°C, then rinsed in 75% alcohol two or three times to remove chlorophyll (Jefferson et al., 1987). The tissues of positive transgenic plants would show blue color.

#### Herbicide (glufosinate) painting

Half (along the midrib) of a leaf (the upper surface) was painted with 135 mg/L glufosinate using a swab, while a black line was drawn on the other half leaf to mark it as control. About seven days later, if the half leaf with glufosinate treatment is same as the control, the plant is tolerant to herbicide and therefore positive.

#### PCR assay

Total genomic DNA was extracted using the CTAB method (Paterson et al., 1993). Gene specific primers were designed by NCBI Primer-BLAST (https://www.ncbi.nlm.nih.gov/tools/primer-blast/), and were used to amplify the 1812 bp *GUS* fragment and 428 bp *bar* fragment (Table S2). The PCR condition was set as the following: one cycle at 95°C for 3 min, followed by 35 cycles of 95°C for 30 s, 56°C for 30 s, 72°C for 1 min 50 s (*GUS*), or 30 s (*bar*), and a final extension at 72°C for 5 min. The amplified PCR products were visualized and photographed using the gel imaging system after electrophoresis on a 0.8% (w/v) agarose gel containing 120 ul/L ethidium bromide.

#### Libertylink® strip *(bar)* test

Leaf tissue was put in an Eppendorf tube and grounded with 0.5 ml extraction buffer by a pestle. Then a LibertyLink® strip was inserted into the tube and waited for 10 min. The appearance of a control line indicates the strip is functional and the second line (test line) indicates the sample is positive.

#### Analysis of gene copy number by absolute quantification PCR

The copy number of *bar* gene integrated in the soybean genomic DNA was estimated by the ratio of exogenous target gene (*bar*)/internal reference gene (*lectin*), X_0_/R_0_, which could be calculated according to the formula: X_0_/R_0_ = 10^[(Ct,X−IX)/SX]−[(Ct,R−IR)/SR]^ (Weng et al., 2004), where C_*t,X*_ and C_*t,R*_ represents the Ct value of target gene (*bar*) and reference gene (*lectin*), respectively; IX and SX is the intercept and slope of the standard curve of the target gene, respectively; IR and SR is the intercept and slope of the standard curve of the reference gene, respectively. The stand curve was obtained by plotting the logarithms of the template DNA copy number (X axis) against the Ct values (Y axis) using a series of DNA template dilutions, where the template DNA copy number = avogadro constant (6.02 × 10^23^) × concentration of template DNA (g/ml)/the relative molecular mass of template DNA (g/mol), and the Ct values were obtained by absolute quantification PCR. The standard curves of the exogenous *bar* gene (on the positive plasmid) and the internal *lectin* gene (on the genomic DNA) were established respectively (Sambrook and Russell, 2001). The size of positive plasmid we used in this study is 11,622 bp, and the genome size of soybean is 1,150 Mb (Schmutz et al., 2010). Because two soybean varieties were used for transformation, the series dilutions of genomic DNA from the control plants of both Jack Purple and Tianlong 1, as well as the positive plasmid were made respectively to generate the standard curves.

Quantification PCR was performed on the LightCycler 480 (Roche, USA) to obtain the Ct values of the exogenous target *bar* gene and the internal reference *lectin* gene (Qiu et al., 2012). The 20 μl qRT-PCR reaction mixture contained the following components: 1 μl (100 ng) template DNA, 10 μl 2 × SYBR® Premix Taq™, 0.4 μl (20 μM) of the forward and reverse primers (Table S2), and 8.2 μl of sterile ddH_2_O. The PCR condition was set as one cycle at 95°C for 3 min, followed by 40 cycles of 95°C for 10 s, 55°C for 15 s, and 72°C for 15 s. Each sample was repeated three times.

### Determination of *Agrobacterium* infection efficiency, rate of shoot elongation and transformation efficiency

The *Agrobacterium* infection efficiency was estimated by the rate of GUS transient expression in soybean cotyledonary explants. The rate of GUS transient expression (%) = (The number of cotyledonary explants in blue color/total number of cotyledonary explants for staining) × 100%. The rate of shoot elongation (%) = (The number of elongated shoots with heights ≥ 3 cm/the number of infected explants) × 100%. The transformation efficiency (%) = (The number of positive transgenic plants / the number of infected explants) × 100%.

### Statistical analysis

SAS 9.2 software was used for statistical analysis. Differences between two groups were analyzed by student's *t*-test, while differences among multiple treatments were analyzed using Duncan's multiple range test. GraphPad Prism, Microsoft Excel, and PowerPoint were used to generate graphs.

## Results

### The effect of DTT on the transient GUS expression in soybean explants

**Figure 3:**
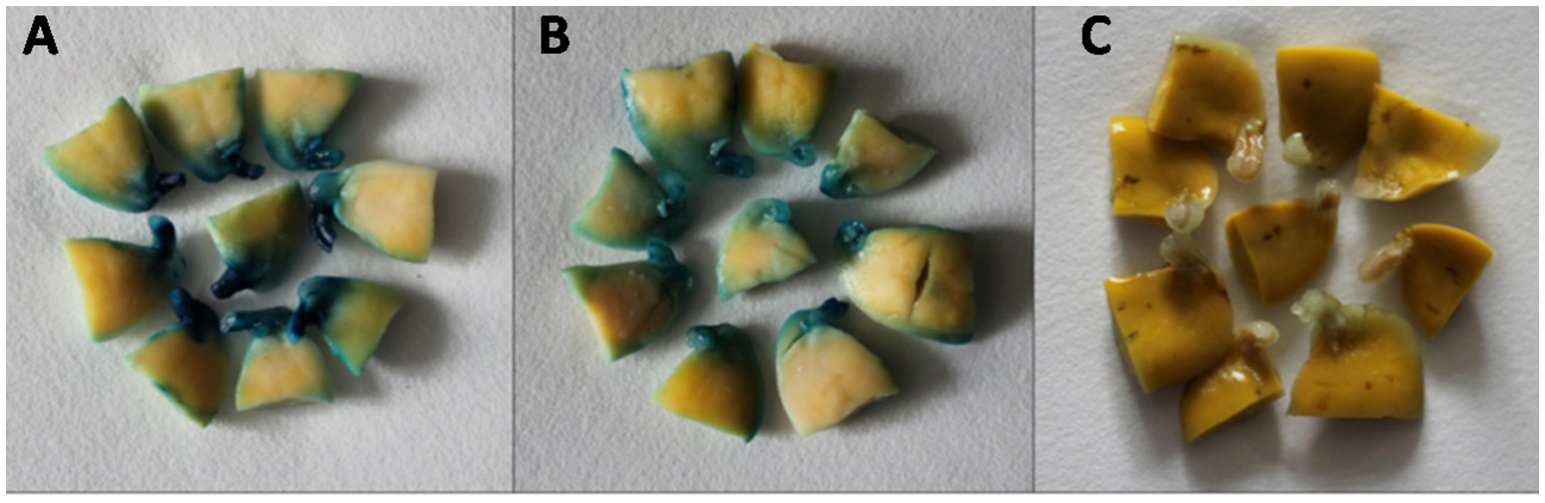
**Classification standards of GUS staining. (A)** Strong, with deep dyeing in the cotyledonary explants. **(B)** Weak, with light dyeing in the cotyledonary explants. **(C)** None, with no dyeing in the cotyledonary explants.

**Figure 4:**
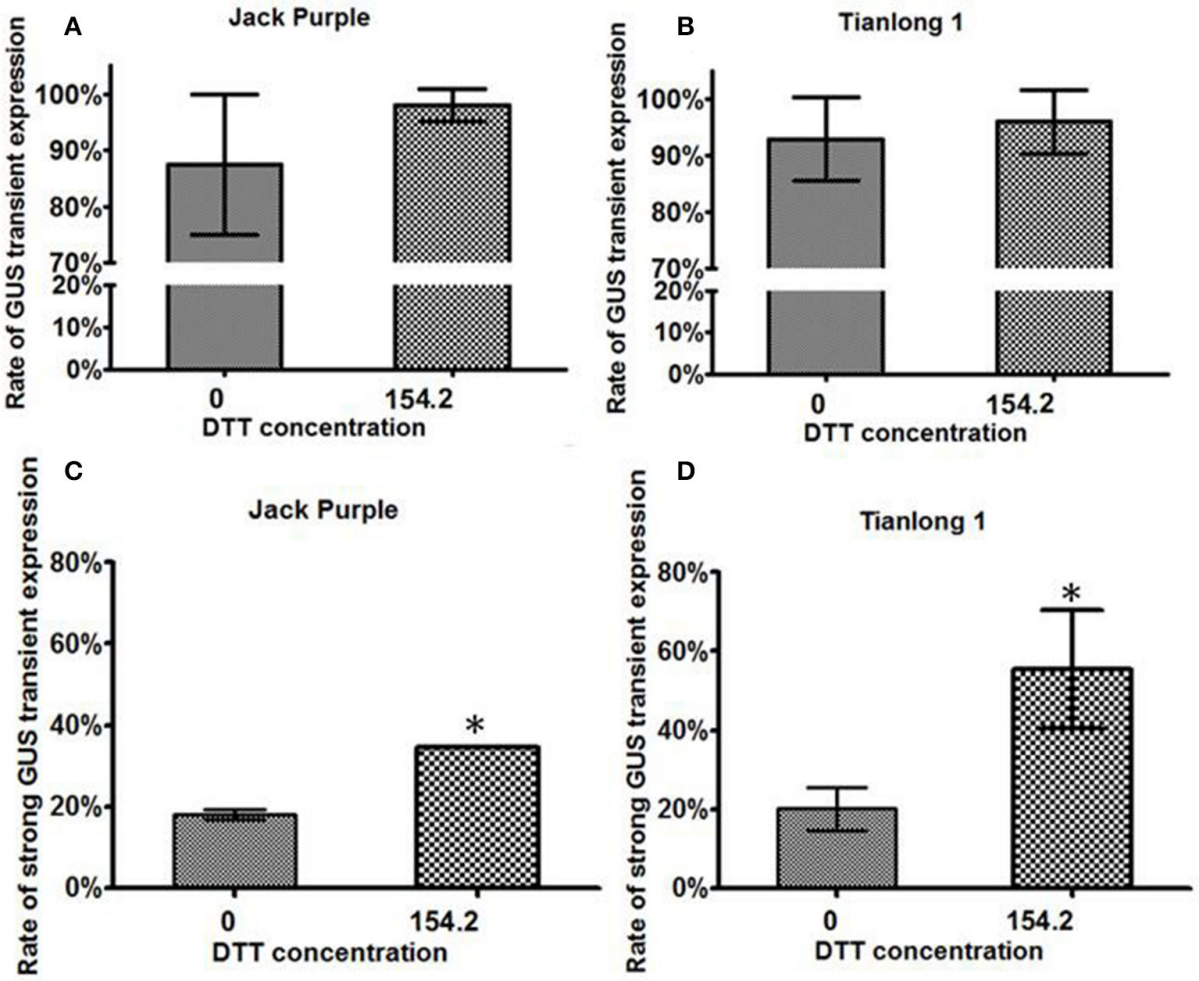
**The effect of DTT on the transient GUS expression in soybean cotyledonary explants. (A)** Rate of total GUS transient expression in Jack Purple explants. **(B)** Rate of total GUS transient expression in Tianlong 1 explants. **(C)** Rate of strong GUS transient expression in Jack Purple explants. **(D)** Rate of strong GUS transient expression in Tianlong 1 explants. Results are expressed as mean ± standard error. Fifty explants were stained for each treatment and the experiments were repeated twice. ^*^ above bars indicates significant difference between 0 and 154.2 mg/L DTT at 0.05 level by student's *t*-test. Rate of total GUS transient expression (%) = (The number of explants with deep and weak dyeing / Total number of explants for staining) × 100%. Rate of strong GUS transient expression (%) = (The number of explants with deep dyeing/Total number of explants for staining) × 100%.

The rate of GUS transient expression in the cotyledonary explants reflects the infection efficiency of *Agrobacterium*. In order to see the effect of the antioxidant DTT on the transient GUS expression in explants, 0 or 154.2 mg/L DTT was added to the re-suspended infection medium after collecting the *Agrobacterium* when OD_650_ = 0.6. The half-seed explants from 1-d imbibition were infected by the *Agrobacterium* suspension medium with or without DTT, and subjected to GUS staining after 5 days of co-cultivation. According to the GUS staining intensity, the explants were divided into three categories: strong, weak, and none (Figure 3). The rate of total transient GUS expression in the cotyledonary explants after adding DTT in re-suspended infection medium was increased by 13 and 3% for Jack Purple and Tianlong 1, respectively (Figures 4A,B), compared with the control (without DTT), and the rate of strong GUS transient expression was significantly (*P* < 0.05, student's *t*-test) increased by 16 and 35% for Jack Purple and Tianlong 1, respectively (Figures 4C,D).

### The effect of *Agrobacterium* concentration on the transient GUS expression in explants

To determine the optimal concentration of *Agrobacterium* for infection, the *Agrobacterium* at three different OD_650_ (0.6, 0.8, 1.0) during logarithmic growth stage (Figure S1) were collected, respectively, and re-suspended in the liquid CCM containing 154.2 mg/L DTT, then co-cultured with soybean half-seed explants (from 1-d imbibition) for 5 days. The explants were stained after 5 days of co-cultivation.

**Figure 5:**
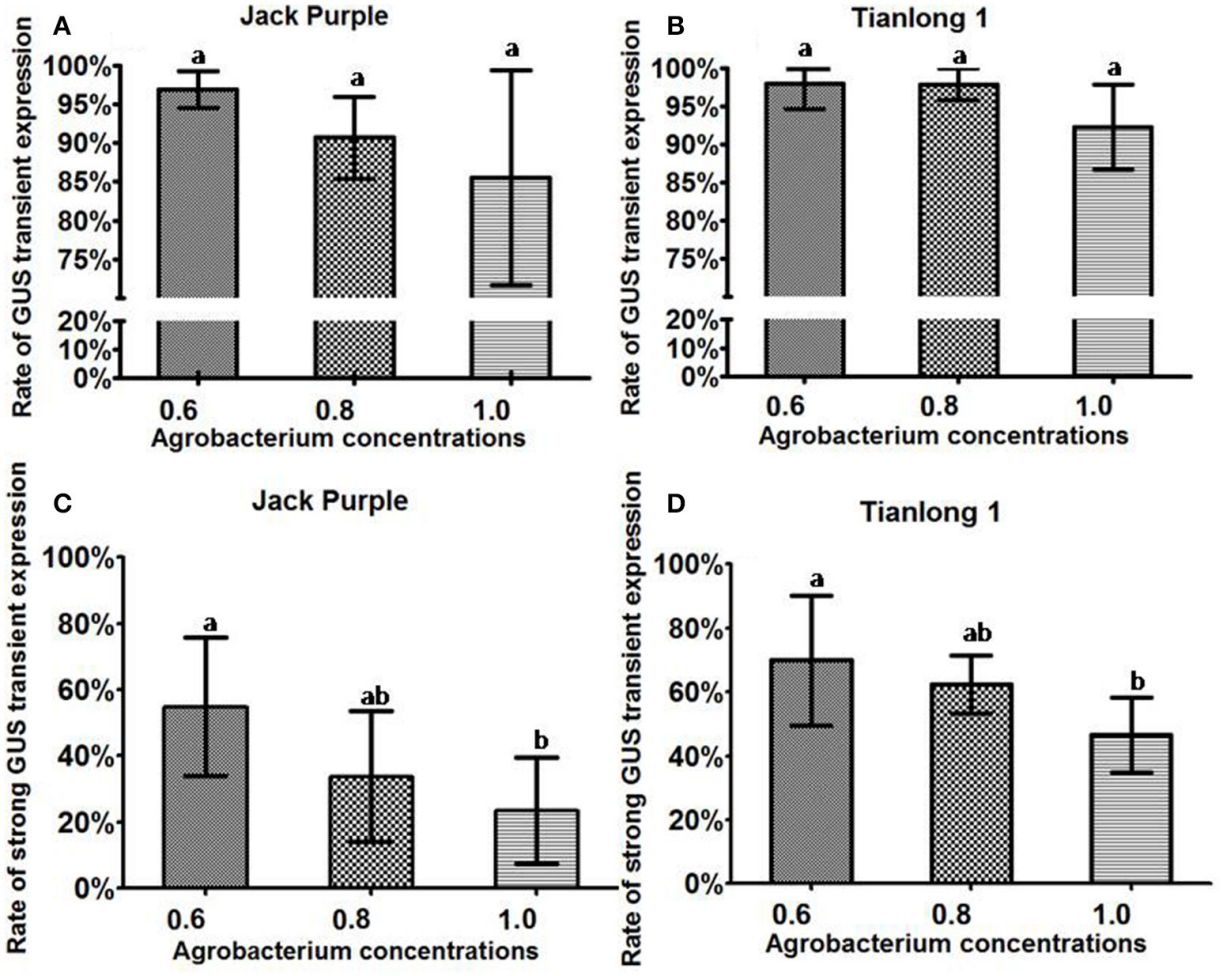
**The effect of ***Agrobacterium*** concentration on the transient GUS expression in soybean cotyledonary explants. (A)** Rate of total GUS transient expression in the cotyledonary explants of Jack Purple. **(B)** Rate of total GUS transient expression in the cotyledonary explants of Tianlong 1. **(C)** Rate of strong GUS transient expression in the cotyledonary explants of Jack Purple. **(D)** Rate of strong GUS transient expression in the cotyledonary explants of Tianlong 1. Results are expressed as mean ± standard error. Fifty explants were stained for each treatment and the experiments were repeated five times. Means with the same letter above bars are not significantly different at 0.05 level according to Duncan's multiple range test. Rate of total GUS transient expression (%) = (The number of explants with deep and weak dyeing/Total number of explants for staining) × 100%. Rate of strong GUS transient expression (%) = (The number of explants with deep dyeing/Total number of explants for staining) × 100%.

The rate of total GUS transient expression in the cotyledonary explants of both soybean varieties (Jack Purple and Tianlong 1) was not significantly different among the concentrations of OD_650_ = 0.6, 0.8, and 1.0 (Figures 5A,B). However, for both soybean varieties, the rate of strong GUS transient expression was significantly (*P* < 0.05, Duncan's multiple range test) higher when using the concentration of OD_650_ = 0.6 than OD_650_ = 1, and slightly higher than OD_650_ = 0.8 (Figures 5C,D). Therefore, we conclude that the optimal concentration of *Agrobacterium* for infection is OD_650_ = 0.6.

### The effect of different explants on the transient GUS expression

**Figure 6:**
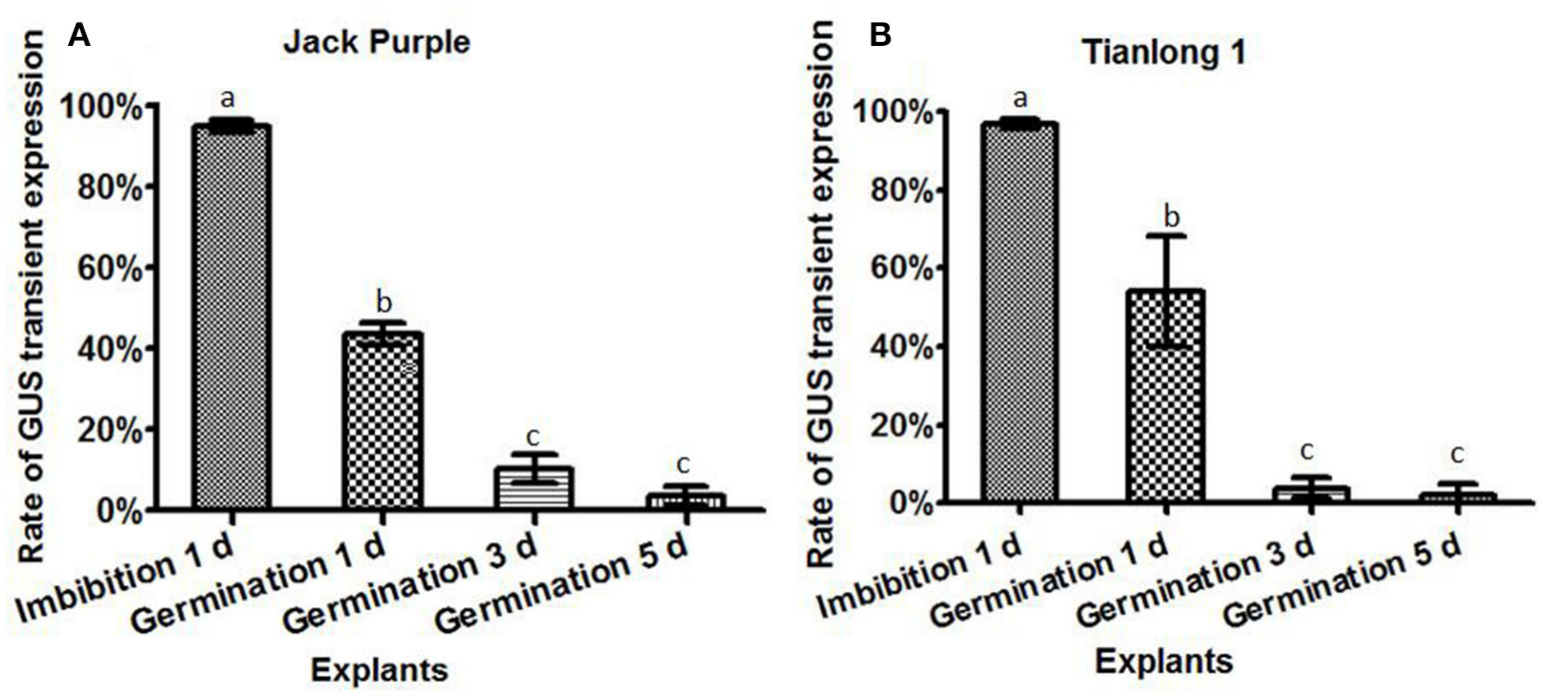
**The effect of different explants on the transient GUS expression**. The cotyledonary explants were obtained by four different methods, including imbibition for 1 d, germination for 1, 3, and 5 d. **(A)** Rate of total GUS transient expression in the cotyledonary explants of Jack Purple. **(B)** Rate of total GUS transient expression in the cotyledonary explants of Tianlong 1. The results are expressed as mean ± standard error. Fifty explants were stained for each treatment and the experiments were repeated twice. Means with the same letter above bars are not significantly different at 0.05 level according to Duncan's multiple range test. Rate of total GUS transient expression (%) = (The number of explants with deep and weak dyeing/Total number of explants for staining) × 100%.

The *Agrobacterium* infection efficiencies were then compared among four different explants prepared by 1-d imbibition, 1-d germination, 3-d germination, and 5-d germination, respectively, using two soybean varieties of Jack Purple and Tianlong 1. The *Agrobacterium* was collected at OD_650_ = 0.6, and re-suspended in the liquid CCM containing 154.2 mg/L DTT to infect above four different explants, respectively. After 5 days of co-cultivation, the explants were subjected to GUS staining. The result showed that the highest rate of total GUS transient expression in the cotyledonary explants was achieved when using the half-seed explants from 1-d imbibition for both soybean varieties, 95 and 97% in Jack Purple and Tianlong 1, respectively, which was significantly (*P* < 0.05, Duncan's multiple range test) higher than the other three types of explants (Figure 6).

### The effect of co-cultivation time on transient GUS expression in soybean explants

**Figure 7:**
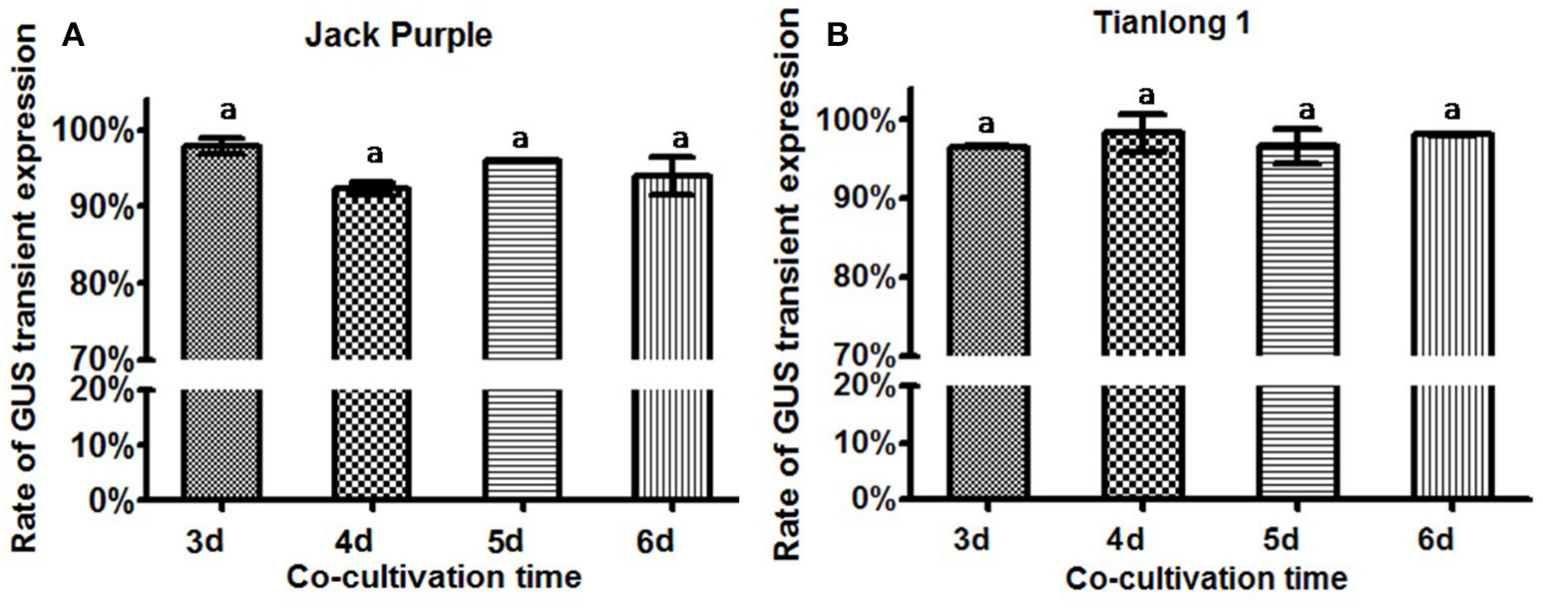
**The effect of co-cultivation time on transient GUS expression in soybean explants. (A)** Rate of total GUS transient expression in the cotyledonary explants of Jack Purple. **(B)** Rate of total GUS transient expression in the cotyledonary explants of Tianlong 1. Results are expressed as mean ± standard error. Fifty explants were stained for each treatment and the experiments were repeated twice. Means with the same letter above bars are not significantly different at 0.05 level according to Duncan's multiple range test. Rate of total GUS transient expression (%) = (The number of explants with deep and weak dyeing/Total number of explants for staining) × 100%.

The *Agrobacterium* was collected at OD_650_ = 0.6, and re-suspended in the liquid CCM containing 154.2 mg/L DTT to infect the soybean half-seed explants from 1-d imbibition. After 3, 4, 5, or 6 days of co-cultivation, the explants were subjected to GUS staining. The results showed that the transient GUS expression rates after four different co-cultivation time were not significantly different (Figure 7). However, the explants were almost yellow when co-cultivated for 3 or 4 days but turned green after 5 days (Figure S2). Therefore, 5-d co-cultivation is chosen when the explants have a strong vitality.

### Rate of transient GUS expression in different soybean varieties

**Figure 8:**
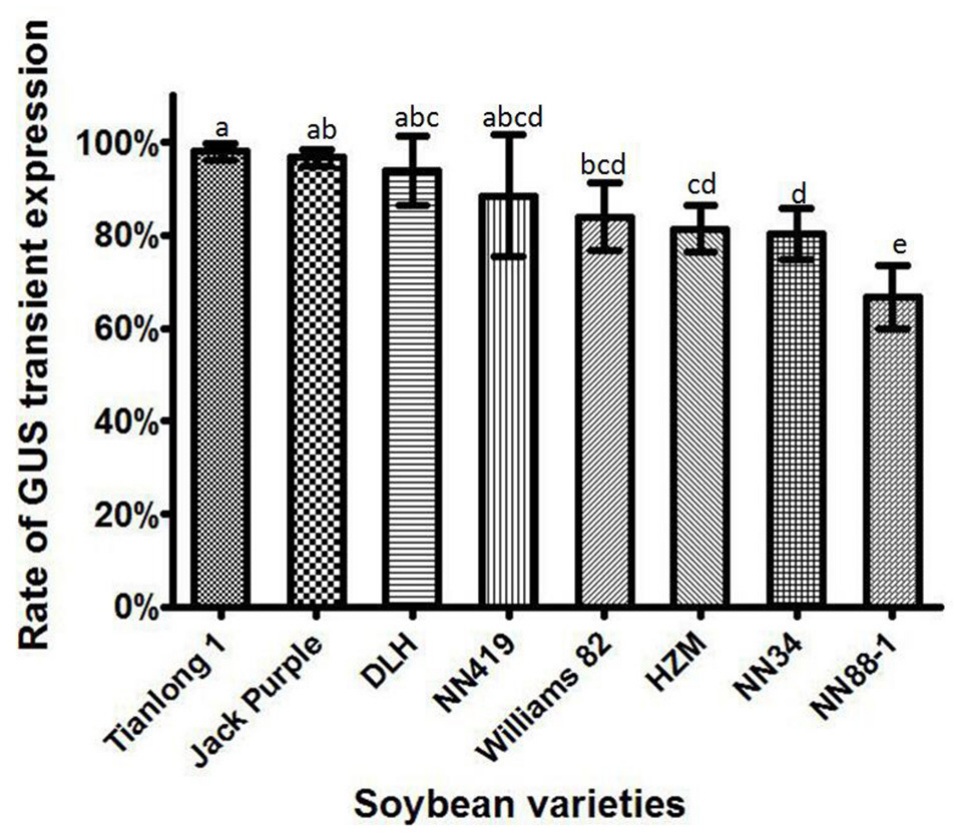
**The transient GUS expression in the cotyledonary explants of different soybean varieties**. Results are expressed as mean ± standard error. Fifty explants were stained for each soybean variety in every replication and the experiments were repeated three times. Means with the same letter above bars are not significantly different at 0.05 level according to Duncan's multiple range test. Rate of total GUS transient expression (%) = (The number of explants with deep and weak dyeing/Total number of explants for staining) × 100%.

Eight soybean varieties, including Tianlong 1, Jack Purple, DLH, NN419, Williams 82, HZM, NN34, and NN88-1, were used to compare the soybean genotype effect on the *Agrobacterium* infection efficiency, with all the other factors optimized (which was collecting the *Agrobacterium* when OD_650_ = 0.6, then re-suspended in the liquid CCM with 154.2 mg/L DTT to infect the half-seed explants from 1-d imbibition, and co-cultured for 5 days). The results showed that the rate of transient GUS expression in Tianlong 1 and Jack Purple was significantly (*P* < 0.05, Duncan's multiple range test) higher than in HZM, NN34, and NN88-1 (Figure 8). We chose Tianlong 1 and Jack Purple in further experiments because of their higher rate of transient GUS expression.

### The effect of different concentration combinations of GA_3_ and IAA on the rate of shoot elongation and transformation efficiency in soybean

**Figure 9:**
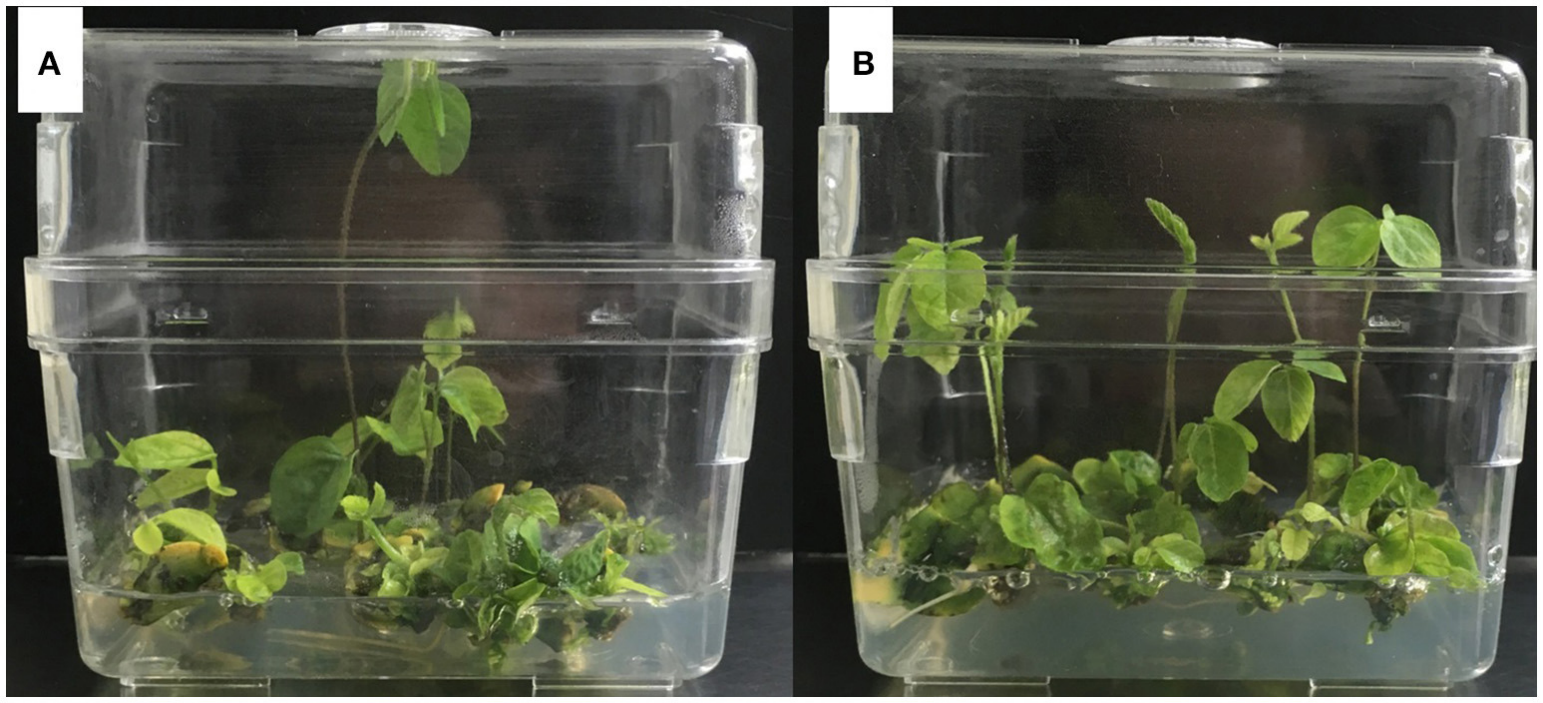
**The explants of soybean variety Jack Purple in the shoot elongation medium. (A)** Soybean explants on SEM with 0.5 mg/L GA_3_ and 0.1 mg/L IAA. **(B)** Soybean explants on SEM with 1.0 mg/L GA_3_ and 0.1 mg/L IAA.

GA_3_ and IAA are important phytohormones to regulate plant growth. GA_3_ promotes stem elongation while IAA mainly promotes cell elongation (Ji and Yang, 2002). Optimum phytohormone concentration is a key factor to improve the regeneration efficiency and transformation efficiency of plants. Based on the original concentration of GA_3_ and IAA (0.5 mg/L GA_3_ and 0.1 mg/L IAA) in SEM (Paz et al., 2006), we used three levels of GA_3_ concentration (0.5, 1.0, and 1.5 mg/L) and two levels of IAA concentration (0.1 and 0.2 mg/L) in the experimental design, which would be six combinations (Table 1). The rate of shoot elongation and transformation efficiency in Jack Purple and Tianlong 1 were calculated respectively (Table 1). The results showed that the highest rate of shoot elongation in Jack Purple was 33.54% when 1.0 mg/L GA_3_ and 0.1 mg/L IAA were added in SEM, which was significantly (*P* < 0.05, Duncan's multiple range test) higher than the original concentration combination of GA_3_ and IAA (0.5 mg/L GA_3_ and 0.1 mg/L IAA) in SEM (Table 1, Figure 9). Similar results were found for Tianlong 1. The highest rate of shoot elongation in Tianlong 1 was 26.08% when 1.0 mg/L GA_3_ and 0.1 mg/L IAA were added in SEM (Table 1). The transformation efficiencies in Jack Purple and Tianlong 1 were 7.32 and 10.01% when SEM contained 1.0 mg/L GA_3_ and 0.1 mg/L IAA, which were higher than 5.00 and 4.28% when SEM contained 0.5 mg/L GA_3_ and 0.1 mg/L IAA, respectively (Table 1). These results suggest that the optimal concentration combination of GA_3_ and IAA (1.0 mg/L GA_3_ and 0.1 mg/L IAA) improved the rate of shoot elongation and transformation efficiency in soybean varieties of Jack Purple and Tianlong 1.

**Table 1:** **The effect of combination of different GA_**3**_ and IAA concentrations on the rate of shoot elongation and transformation efficiency in soybean**.

**Soybean variety**	**GA_3_ concentration (mg/L)**	**IAA concentratio (mg/L)n**	**Rate of shoot elongation (%)**	**Transformation efficiency (%)**
Jack Purple	0.5	0.1	16.11 ± 0.05^b^	5.00 ± 0.03^a^
	0.5	0.2	13.70 ± 0.02^b^	4.57 ± 0.01^a^
	1.0	0.1	33.54 ± 0.03^a^	7.32 ± 0.01^a^
	1.0	0.2	17.42 ± 0.01^b^	5.26 ± 0.01^a^
	1.5	0.1	11.49 ± 0.01^b^	5.41 ± 0.03^a^
	1.5	0.2	13.39 ± 0.05^b^	5.36 ± 0.01^a^
Tianlong 1	0.5	0.1	14.75 ± 0.01^ab^	4.28 ± 0.00^a^
	0.5	0.2	10.60 ± 0.01^b^	1.76 ± 0.02^a^
	1.0	0.1	26.08 ± 0.07^a^	10.01 ± 0.03^a^
	1.0	0.2	9.80 ± 0.04^b^	3.53 ± 0.02^a^
	1.5	0.1	19.56 ± 0.05^ab^	4.49 ± 0.03^a^
	1.5	0.2	14.40 ± 0.03^ab^	5.69 ± 0.04^a^

*The results are expressed as mean ± standard error. Eighty explants were infected by Agrobacterium for each combination/treatment and the experiments were repeated twice. The concentrations of glufosinate for selection were 5 mg/L and 3 mg/L in SIM and SEM, respectively. The numbers of elongated shoots (height ≥ 3 cm) were recorded during SEM stage. For each soybean variety, means with the same letter are not significantly different at 0.05 level according to Duncan's multiple range test. Rate of shoot elongation (%) = (The number of elongated shoots/the number of infected explants) × 100%. Transformation efficiency (%) = (The number of the positive plants/the number of infected explants) × 100%*.

### The effect of AgNO_3_ and Vc on the rate of shoot elongation and transformation efficiency in soybean

Tissue browning is another factor affecting transformation efficiency. 88-1 is one of the soybean varieties that encountered more serious tissue browning. Therefore, we added AgNO_3_ (5, 10, or 15 mg/L) to SIM and SEM (with 0.5 mg/L GA_3_ and 0.1 mg/L IAA in SEM) during tissue culturing 88-1 explants. We did not observe alleviation of the tissue browning after adding AgNO_3_. However, the shoot elongation rate increased after adding different concentrations of AgNO_3_, and the transformation efficiency in 88-1 improved from 3.17 to 5.50% after adding 15 mg/L AgNO_3_ (Table S3).

Therefore, we further investigated the effect of adding 15 mg/L AgNO_3_ in SIM and SEM (with 0.1 mg/L IAA in SEM) using the other two soybean varieties of Jack Purple and Tianlong 1. Because doubling GA_3_ concentration in SEM improved the rate of shoot elongation (Table 1), we investigated the effect of AgNO_3_ under two levels of GA_3_ concentration in SEM. In Jack Purple, the results showed that the rate of shoot elongation and transformation efficiency decreased when adding 15 mg/L AgNO_3_ in SIM and SEM (Table 2), and abnormal leaves were observed on Jack Purple after adding 15 mg/L AgNO_3_ in the medium (Figure S3). In Tianlong 1, the rate of shoot elongation increased from 14.75 to 19.47% at the level of 0.5 mg/L GA_3_ and from 26.08 to 31.82% at the level of 1.0 mg/L GA_3_. However, adding 15 mg/L AgNO_3_ in SIM and SEM had little effect on the transformation efficiency (Table 2).

**Table 2:** **The effect of AgNO_**3**_ on the rate of shoot elongation and transformation efficiency in soybean**.

**Soybean variety**	**GA_3_ concentration (mg/L)**	**AgNO_3_ concentration (mg/L)**	**Rate of shoot elongation (%)**	**Transformation efficiency (%)**
Jack Purple	0.5	0	16.11 ± 0.05^b^	5.00 ± 0.03^a^
	0.5	15	14.44 ± 0.00^b^	3.89 ± 0.02^a^
	1	0	33.54 ± 0.03^a^	7.32 ± 0.01^a^
	1	15	26.22 ± 0.02^ab^	6.71 ± 0.01^a^
Tianlong 1	0.5	0	14.75 ± 0.01^a^	4.28 ± 0.00^a^
	0.5	15	19.47 ± 0.03^a^	5.44 ± 0.03^a^
	1	0	26.08 ± 0.07^a^	10.01 ± 0.03^a^
	1	15	31.82 ± 0.00^a^	9.09 ± 0.01^a^

*The results are expressed as mean ± standard error. Eighty explants were infected by Agrobacterium for each combination/treatment and the experiments were repeated twice. The concentrations of glufosinate for selection were 5 mg/L and 3 mg/L in SIM and SEM, respectively. The numbers of elongated shoots (height ≥ 3 cm) were recorded during SEM stage. For each soybean variety, means with the same letter are not significantly different at 0.05 level according to Duncan's multiple range test. Rate of shoot elongation (%) = (The number of elongated shoots/the number of infected explants) × 100%. Transformation efficiency (%) = (The number of the positive plants / the number of infected explants) × 100%*.

Previous studies showed that adding 200 mg/L Vc in the medium can prevent tissue browning in *Bromeliaceae* (Peng et al., 2007) and promote rooting in *Limonium* (Xu et al., 2010). Therefore, we added 200 mg/L Vc to SIM and SEM (with 0.1 mg/L IAA in SEM) to see if Vc can prevent browning and improve the rate of shoot elongation and transformation efficiency in Jack Purple and Tianlong 1. For Jack Purple, the rate of shoot elongation increased from 16.11 to 23.17%, and the transformation efficiency increased from 5.00 to 9.15% after adding 200 mg/L Vc in SIM and SEM when GA_3_ concentration was 0.5 mg/L in SEM. When the GA_3_ concentration was 1 mg/L in SEM, the rate of shoot elongation was reduced but the transformation efficiency increased slightly (Table 3). For Tianlong 1, the rate of shoot elongation and transformation efficiency was slightly improved after adding 200 mg/L Vc in SIM and SEM when GA_3_ concentration was 0.5 mg/L in SEM. However, at 1 mg/L GA_3_ in SEM, the rate of shoot elongation and transformation efficiency decreased after adding 200 mg/L Vc in SIM and SEM. These results suggested that the tissue culture medium should be optimized specifically for different soybean varieties.

**Table 3:** **The effect of Vc on the rate of shoot elongation and transformation efficiency in soybean**.

**Soybean variety**	**GA_3_ concentration (mg/L)**	**Vc concentration (mg/L)**	**Rate of shoot elongation (%)**	**Transformation efficiency (%)**
Jack Purple	0.5	0	16.11 ± 0.05^b^	5.00 ± 0.03^a^
	0.5	200	23.17 ± 0.04^ab^	9.15 ± 0.02^a^
	1	0	33.54 ± 0.03^a^	7.32 ± 0.01^a^
	1	200	28.05 ± 0.04^ab^	8.54 ± 0.02^a^
Tianlong 1	0.5	0	14.75 ± 0.01^a^	4.28 ± 0.00^a^
	0.5	200	17.73 ± 0.02^a^	4.55 ± 0.00^a^
	1	0	26.08 ± 0.07^a^	10.01 ± 0.03^a^
	1	200	19.09 ± 0.02^a^	4.55 ± 0.01^a^

*The results are expressed as mean ± standard error. Eighty explants were infected by Agrobacterium for each combination/treatment and the experiments were repeated twice. The concentrations of glufosinate for selection were 5 mg/L and 3 mg/L in SIM and SEM, respectively. The numbers of elongated shoots (height ≥ 3 cm) were recorded during SEM stage. For each soybean variety, means with the same letter are not significantly different at 0.05 level according to Duncan's multiple range test. Rate of shoot elongation (%) = (The number of elongated shoots/the number of infected explants) × 100%. Transformation efficiency (%) = (The number of the positive plants/the number of infected explants) × 100%*.

### Confirmation of positive transgenic soybean plants

The positive T_0_ transgenic soybean plants were first identified by GUS staining of young leaves during rooting stage. A total of 20 positive transgenic plants were randomly picked for further confirmation using the other four different methods, including GUS staining after transplanting, PCR amplification of exogenous gene, herbicide (glufosinate) painting, and LibertyLink® strip detection. The results (Table 4) showed that all methods were consistent except that GUS staining after transplanting missed four positive transgenic plants. Therefore, GUS staining during rooting stage is recommended first since this method can identify positive transgenic plants as soon as possible, and herbicide painting would be an easy and reliable method to confirm the results.

**Table 4:** **Comparison of different methods to detect positive transgenic soybean plants at T_**0**_ generation**.

**Method**	**Number of positive plants/Total plants**	**Time after shoot elongation**	**Time needed for testing**
GUS staining during rooting	20/20	1 day	Overnight
GUS staining after transplanting	16/20	2 weeks	Overnight
PCR amplification of *GUS* gene	20/20	3–4 weeks	2 h
Glufosinate painting on leaves	20/20	4 weeks	7 days
LibertyLink® strip detection	20/20	4 weeks	10 min

**Figure 10:**
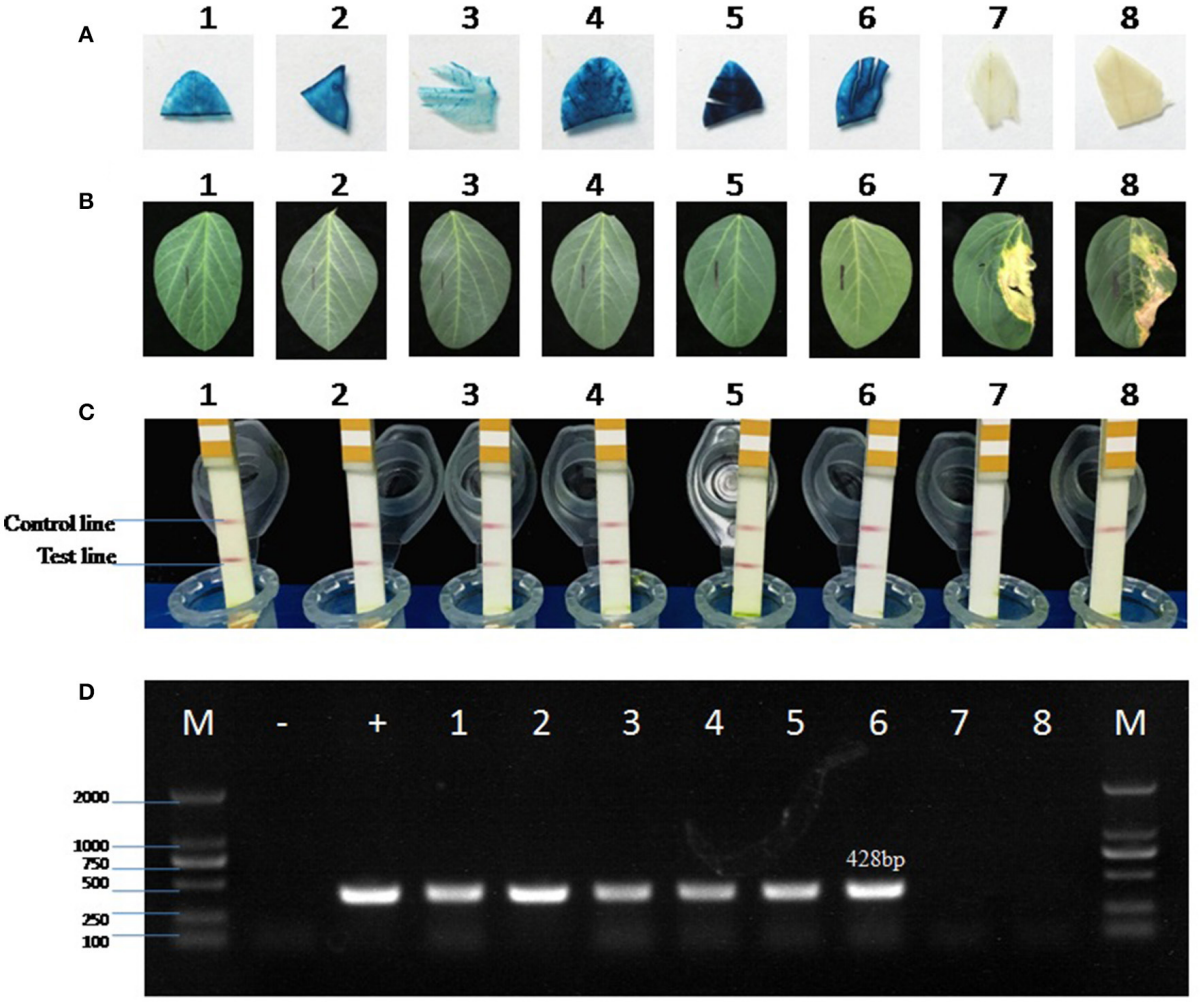
**Detection of the positive transgenic soybean plants. (A)** GUS staining of soybean leaves. **(B)** Herbicide (glufosinate) painting on soybean leaves. The left was marked by a black line as control, and the right was painted by 135 mg/L glufosinate. **(C)** LibertyLink® strip detection. The first line is control line, and the second line is test line. **(D)** PCR amplification of the 428-bp *bar* gene fragment using negative control (−, ddH_2_O), positive control (+, plasmid pTF102), or soybean genomic DNA as the template. M, 2000 bp DNA marker. 1–3, positive transgenic soybean plants in Jack Purple background. 4–6, positive transgenic soybean plants in Tianlong 1 background. 7, negative control of Jack Purple. 8, negative control of Tianlong 1.

The positive T_1_ transgenic soybean plants were also confirmed by four different methods (Figure 10). The copy number of *bar* gene was analyzed by absolute quantitative PCR using *lectin* as the reference gene (Qiu et al., 2012). The melting curves of *bar* and *lectin* genes showed specific amplification (Figure S4). The equation for the standard curve of the endogenous reference gene *lectin* in Jack Purple and Tianlong 1 was *y* = −3.483x + 35.33 (*R*^2^ = 0.995), and *y* = −3.167x + 36.49 (*R*^2^ = 0.986), respectively; while the equation for the standard curve of *bar* gene in plasmid was *y* = −3.116x + 40.78 (*R*^2^ = 0.984). Then the ratio of exogenous gene (*bar*) and reference gene (*lectin*) was calculated. Because *lectin* is a single gene in soybean genome (Vodkin et al., 1983), so the ratio of exogenous gene (*bar*) and reference gene (*lectin*) represents the copy of the exogenous gene (*bar*). The results showed that transgenic soybean line 3 and line 6 had one copy, line 2, and line 4 had two copies, line 1 and line 5 had three copies of *bar* gene (Table 5).

**Table 5:** **The copy number of ***bar*** gene in T_**1**_ transgenic soybean plants**.

**Sample^a^**	**C^b^_t,X_**	**C^c^_t,R_**	**Ratio of *bar*/*lectin* (X_0_/R_0_)^d^**	**Copy number of *bar* gene**
1	26.94 ± 0.12	20.52 ± 0.00	3.10	3
2	26.24 ± 0.39	20.78 ± 0.56	1.55	2
3	26.74 ± 0.11	20.17 ± 0.21	1.42	1
4	25.51 ± 0.24	21.58 ± 0.09	1.56	2
5	24.39 ± 0.17	21.52 ± 0.23	3.42	3
6	26.54 ± 0.08	21.85 ± 0.15	0.88	1

a *1–3, positive T_1_ transgenic soybean plants in Jack Purple background. 4–6, positive T_1_ transgenic soybean plants in Tianlong 1 background*.

b *C_t,X_ represents the Ct value of the exogenous target gene (bar)*.

c *C_t,R_ represents the Ct value of the internal reference gene (lectin)*.

d *X_0_/R_0_ is the ratio of initial amount of bar/lectin. X_0_/R_0_ = 10^[(Ct, X−IX)/SX]−[(Ct, R−IR)/SR]^, where IX and SX is the intercept and slope of the standard curve of the target bar gene, respectively, and IR and SR is the intercept and slope of the standard curve of the reference lectin gene, respectively*.

## Discussion

Since the cotyledonary nodes were used as explants to obtain the first transgenic soybean plant by *Agrobacterium*-mediated transformation (Hinchee et al., 1988), this method has been modified and an improved transformation efficiency of 3.8% (on average) was achieved (Paz et al., 2006). However, this efficiency is still low to sufficiently screen enough transformation events for molecular breeding or gene function studies. Therefore, we try to further improve the transformation efficiency by increase the *Agrobacterium* infection efficiency and the rate of shoot elongation in soybean.

When the *Agrobacterium* concentration is too high to infect the explants, it is hard to wash the *Agrobacterium* away from the explants and will lead to *Agrobacterium* contamination. But if the concentration of *Agrobacterium* is too low, the infection ability is weak (Zhong, 2007). Therefore, it is better to choose a relatively low concentration with high infection ability. In this study, we found that OD_650_ = 0.6 is the optimum concentration for infection. In addition, other factors affecting *Agrobacterium* infection efficiency were also investigated by estimation of the rate of GUS transient expression in soybean cotyledonary explants, including soybean explants, *Agrobacterium* suspension medium, and co-cultivation time. An infection efficiency of over 96% was achieved by collecting the *Agrobacterium* at a concentration of OD_650_ = 0.6, then re-suspended in liquid CCM containing 154.2 mg/L DTT to infect the half-seed cotyledonary explants (from mature seeds imbibed for 1 day), and co-cultured them for 5 days. Among the eight soybean varieties, higher *Agrobacterium* infection efficiencies were observed for soybean varieties Tianlong 1, Jack Purple, DLH, and NN419 (Figure 8).

It has been reported in other plant spices that the optimum concentration of GA_3_ and IAA in SEM could improve the rate of shoot elongation effectively (Gonbad et al., 2014). We investigated six different concentration combinations of GA_3_ and IAA in SEM, and the highest rate of shoot elongation was achieved (33.54% in Jack Purple and 26.08% in Tianlong 1) when 1.0 mg/L GA_3_ and 0.1 mg/L IAA were added in SEM (Table 1). A previous study showed that a higher elongation rate was observed when adding 0.5 mg/L GA_3_ without IAA in SEM when using the soybean variety Heinong 35 explants (Li et al., 2008a). In another study, the highest shoot elongation rate was obtained by adding 1 mg/L GA_3_ and 0.5 mg/L IAA to SEM for soybean variety Jiyu47 (Sun, 2013). These results suggest that the optimal concentration combination of GA_3_ and IAA in SEM varies among different soybean genotypes.

Plants will generate reactive oxygen species upon the infection of *Agrobacterium*, which leads to cell death and tissue browning. Low concentrations of antioxidants can prevent cell necrosis and improve the transformation efficiency. In this study, the rate of strong GUS transient expression in the cotyledonary explants of Jack Purple and Tianlong 1 increased by 16 and 35%, respectively, after adding the antioxidant DTT in CCM (Figure 4). The previous study showed that adding Vc in the medium can reduce the degree of tissue browning significantly, and the addition of AgNO_3_ can not only reduce tissue browning effectively, but also improve the regeneration efficiency and the number of shoots significantly (Huang et al., 2006). However, in our experiment, the addition of AgNO_3_ or Vc in SIM and SEM did not reduce the browning of the explants. Adding 15 mg/L AgNO_3_ in SIM and SEM improved the rate of shoot elongation and transformation efficiency in 88-1 (Table S3), and slightly increased the shoot elongation rate in Tainlong 1, but reduced the rate of shoot elongation and transformation efficiency in Jack Purple (Table 2). Adding 200 mg/L Vc in SIM and SEM only slightly increased the rate of shoot elongation when GA_3_ was 0.5 mg/L but not at 1 mg/L GA_3_ for Jack Purple and Tianlong 1 (Table 3). Therefore, the effect of AgNO_3_ and Vc on the shoot elongation rate and transformation efficiency would depend on soybean genotype and GA_3_ concentration.

In this study, we used four different methods to detect the positive transgenic plants, including GUS histochemical staining, PCR amplification of exogenous gene, herbicide (glufosinate) painting, and LibertyLink® strip detection. GUS staining during rooting stage is recommended to identify positive transgenic plants, which can reduce labor intensity by eliminating negative plants as soon as possible, and herbicide painting would be an easy and reliable method to confirm the results. GUS staining after transplanting might miss the detection of some positive plants, which is likely due to the fact that older leaves are difficult to get stained.

Southern blot has been a traditional method to detect the copy number of exogenous gene in transgenic plants, which gives us highly precise and intuitive results, but complex operations and large amounts of plant material are required. Absolute quantitative PCR technology provides a new approach to detect the copy number of integrated exogenous gene in transgenic plants (Ingham et al., 2001; Weng et al., 2004), which has many advantages such as lower cost, simple operation, high sensitivity and stability, and has been successfully applied to cotton (Yang, 2012), wheat (Gadaleta et al., 2011), rice (Wei et al., 2011), maize (Yuan et al., 2010), tomato (Wang et al., 2011), and soybean (Qiu et al., 2012). In this study, we used absolute quantitative PCR to detect the copy number of exogenous gene (*bar*) in the T_1_ generation of transgenic soybean plants, and the results showed that the copy number of *bar* gene ranged from one to three. The copy number of exogenous gene in transgenic plants affects the expression level and genetic stability of the exogenous gene. The integration of multiple copies of exogenous DNA into one or more chromosomes might result in low gene expression level, low genetic stability, or even gene silencing (Iyer and Kumpatla, 2000; James et al., 2002). The ideal copy number of target gene in transgenic plants is generally one or two (Tang et al., 2007). In this study, four out of six (67%) transgenic plants contained low copy numbers (one or two), which suggests that the *Agrobacterium*-mediated transformation is a preferred method for soybean transformation.

## Conclusion

In this study, the *Agrobacterium*-mediated transformation efficiency in soybean was improved by increasing both *Agrobacterium* infection efficiency and explant regeneration efficiency. The *Agrobacterium* infection efficiency was more than 96% when collecting the *Agrobacterium* at a concentration of OD_650_ = 0.6, then re-suspended in liquid CCM containing 154.2 mg/L DTT to infect the half-seed cotyledonary explants (from mature seeds imbibed for 1 day), and co-cultured them for 5 days, using the soybean varieties of Jack Purple or Tianlong 1. The shoot elongation rate of Jack Purple and Tianlong 1 increased to 33.54 and 26.08% when 1.0 mg/L GA_3_ and 0.1 mg/L IAA were added to SEM, which is almost twice of the previous shoot elongation rate (16.11 and 14.75% for Jack Purple and Tianlong 1, respectively) with 0.5 mg/L GA_3_ and 0.1 mg/L IAA in SEM. Ultimately, the transformation efficiency was improved from 5.00 to 7.32% and 4.28 to 10.01% for Jack Purple and Tianlong 1, respectively. This study provides an optimized *Agrobacterium*-mediated transformation protocol for soybean varieties of Jack Purple and Tianlong 1, and would be a useful reference for improving transformation efficiencies in other plant species.

## Author contributions

SL, YC, and YLi conceived and designed the experiments. SL, YC, YLiu, TW, QS, and NC performed the experiments. SL, YC, and YLi analyzed the data. SL, YC, and YLi generated the pictures. SL and YLi wrote and revised the manuscript. JG and YLi contributed reagents/materials and interpretation of the results. All authors read, revised and approved the final manuscript.

## Funding

This work was supported by the Key Transgenic Breeding Program of China (2014ZX0801005B), the National Natural Science Foundation of China (31371645), the Fundamental Research Funds for the Central Universities, the Program for Changjiang Scholars and Innovative Research Team in University (PCSIRT13073), the MOE 111 Project (B08025), the Program for MOA Innovative Research Team, and the Program for High-level Innovative and Entrepreneurial Talents in Jiangsu Province.

### Conflict of interest statement

The authors declare that the research was conducted in the absence of any commercial or financial relationships that could be construed as a potential conflict of interest.

## References

[B1] Almqvist C. (2003). Timing of GA4/7 application and the flowering of *Pinus sylvestris* grafts in the greenhouse. Tree Physiol. 23, 413–418. 10.1093/treephys/23.6.413 12642243

[B2] An G. Ebert P. R. Mitra A. Ha S. B. (1989). Binary vectors, in Plant Molecular Biological Manual, eds Gelvin S. B. Schilperoort R. A. Verma D. P. (Dordrecht: Springer Netherlands; Kluwer Academic Publishers), 29–47. 10.1007/978-94-009-0951-9_3

[B3] An X. Wang B. Liu L. Jiang H. Chen J. Ye S.. (2014). Agrobacterium-mediated genetic transformation and regeneration of transgenic plants using leaf midribs as explants in ramie [*Boehmeria nivea* (L.) Gaud]. Mol. Biol. Rep. 41, 3257–3269. 10.1007/s11033-014-3188-4 24488319

[B4] Bailey M. A. Parrott W. A. (1993). Genotype effects on proliferative embryogenesis and plant regeneration of soybean. In Vitro Cell. Dev. Biol. Plant 29, 102–108. 10.1007/BF02632279

[B5] Dan Y. (2008). Biological functions of antioxidants in plant transformation. In Vitro Cell. Dev. Biol. Plant 44, 149–161. 10.1007/s11627-008-9110-9

[B6] Dan Y. H. Armstrong C. L. Dong J. Feng X. R. Fry J. E. Keithly G. E. (2009). Lipoic acid-an unique plant transformation enhancer. In Vitro Cell. Dev. Biol. Plant 45, 630–638. 10.1007/s11627-009-9227-5

[B7] Di R. Purcell V. Collins G. B. Ghabrial S. A. (1996). Production of transgenic soybean lines expressing the bean pod mottle virus coat protein precursor gene. Plant Cell Rep. 15, 746–750. 10.1007/BF00232220 24178163

[B8] Donaldson P. A. Simmonds D. H. (2000). Susceptibility to *Agrobacterium tumefaciens* and cotyledonary node transformation in short-season soybean. Plant Cell Rep. 19, 478–484. 10.1007/s002990050759 30754886

[B9] Dong S. Zhang Y. Lv M. Yang F. Gao Y. Wang H. (2011). Advances problems and break through direction of plant transgenic technique. J. Hebei. Agric. 15, 57–65. 10.3969/j.issn.1088-1631.2011.03.019

[B10] Enríquez Obregó G. A. Vázquez Padrón R. I. Prieto-Samsonov D. L. Riva G. A. D. L. Selman Housein G. (1998). Herbicide-resistant sugarcane (*Saccharum officinarum* L.) plants by Agrobacterium -mediated transformation. Planta 206, 20–27. 10.1007/s004250050369

[B11] Frame B. R. Kan W. (2002). *Agrobacterium tumefaciens*-mediated transformation of maize embryos using a standard binary vector system. Plant Physiol. 129, 13–22. 10.1104/pp.000653 12011333 PMC1540222

[B12] Gadaleta A. Giancaspro A. Cardone M. F. Blanco A. (2011). Real-time PCR for the detection of precise transgene copy number in durum wheat. Cell. Mol. Biol. Lett. 16, 652–668. 10.2478/s11658-011-0029-5 21922222 PMC6275630

[B13] Gamborg O. L. Al E. (1968). Nutrient requirements of suspension cultures of soybean root cells. Exp. Cell Res. 50, 151–158. 10.1016/0014-4827(68)90403-5 5650857

[B14] Gao L. Ding X. Li K. Liao W. Zhong Y. Ren R.. (2015). Characterization of Soybean mosaic virus resistance derived from inverted repeat-SMV-HC-Pro genes in multiple soybean cultivars. Theor. Appl. Genet. 128, 1489–1505. 10.1007/s00122-015-2522-0 25930057

[B15] Ge X. Chu Z. Lin Y. Wang S. (2006). A tissue culture system for different germplasms of indica rice. Plant Cell Rep. 25, 392–402. 10.1007/s00299-005-0100-7 16432631

[B16] Gnasekaran P. Antony J. J. J. Uddain J. Subramaniam S. (2014). Agrobacterium-mediated transformation of the recalcitrant vanda kasem's delight orchid with higher efficiency. Sci. World J. 2014:583934. 10.1155/2014/583934 24977213 PMC4000978

[B17] Gonbad R. A. Rani S. U. Aziz M. A. Mohamad R. (2014). Influence of cytokinins in combination with GA_3_ on shoot multiplication and elongation of tea clone Iran 100 (*Camellia sinensis* (L.) O. Kuntze). Sci. World J. 5, 149–168. 10.1155/2014/943054 PMC3926387 24605069

[B18] Han L. Gai J. Zhang W. (2003). The present study of soybean nutrients. Seed 131, 57–59. 10.3969/j.issn.1001-4705.2003.05.023

[B19] Hinchee M. A. W. Connor-Ward D. V. Newell C. A. Mcdonnell R. E. Sato S. J. Gasser C. S. (1988). Production of transgenic soybean plants using agrobacterium-mediated dna transfer. Nat. Biotechnol. 6, 915–922. 10.1038/nbt0888-915

[B20] Hood E. E. Helmer G. L. Fraley R. T. Chilton M. D. (1986). The hypervirulence of *Agrobacterium tumefaciens* A281 is encoded in a region of pTiBo542 outside of T-DNA. J. Bacteriol. 168, 1291–1592. 10.1128/jb.168.3.1291-1301.1986 3782037 PMC213636

[B21] Hua W. Y. Irving H. R. (2011). Developing a model of plant hormone interactions. Plant Signal. Behav. 6, 494–500. 10.4161/psb.6.4.14558 21406974 PMC3142376

[B22] Huang J. Ma F. Fan J. Li X. Tong J. (2006). *In vitro* plant regeneration with adventitious buds of Zizyphus jujuba leaves. Acta Bot. Boreali-Occidentalia Sinica 26, 942–948. 10.3321/j.issn:1000-4025.2006.05.012

[B23] Ingham D. J. Beer S. Money S. Hansen G. (2001). Quantitative real-time PCR assay for determining transgene copy number in transformed plants. BioTechniques 31, 136–140. 11464506 10.2144/01311rr04

[B24] Ishida Y. Saito H. Ohta S. Hiei Y. Komari T. Kumashiro T. (1996). High efficiency transformation of maize (Zea mays L.) mediated by *Agrobacterium tumefaciens*. Nat. Biotechnol. 14, 745–750. 10.1038/nbt0696-745 9630983

[B25] Iyer L. M. Kumpatla S. P. (2000). Transgene silencing in monocots. Plant Mol. Biol. 43, 323–346. 10.1023/A:1006412318311 10999414

[B26] James V. A. Avart C. Worland B. Snape J. W. Vain P. (2002). The relationship between homozygous and hemizygous transgene expression levels over generations in populations of transgenic rice plants. Theor. Appl. Genet. 104, 553–561. 10.1007/s001220100745 12582658

[B27] Jefferson R. A. Kavanagh T. A. Bevan M. W. (1987). GUS fusions: beta-glucuronidase as a sensitive and versatile gene fusion marker in higher plants. EMBO J. 6, 3901–3907. 3327686 10.1002/j.1460-2075.1987.tb02730.x PMC553867

[B28] Ji L. Yang R. (2002). Rice stem elongation and plant hormones. Chin. Bull. Bot. 19, 109–115. 10.3969/j.issn.1674-3466.2002.01.016

[B29] Kim J. H. Lamotte C. E. Hack E. (1990). Plant regeneration *in vitro* from primary leaf nodes of soybean (Glycine max) seedlings. J. Plant Physiol. 136, 664–669. 10.1016/S0176-1617(11)81341-6

[B30] Kumari U. Vishwakarma R. K. Gupta N. Ruby Shirgurkar M. V. Khan B. M. (2015). Efficient shoots regeneration and genetic transformation of *Bacopa monniera*. Physiol. Mol. Biol. Plants 21, 261–267. 10.1007/s12298-015-0290-6 25964718 PMC4411393

[B31] Letham D. S. (1967). Regulators of cell division in plant tissues: V. A comparison of the activities of zeatin and other cytokinins in five *bioassays*. Planta 74, 228–242. 10.1016/s0040-4020(01)83332-9 24549949

[B32] Li W. Li W. Lv W. Ning H. (2008a). Break through of two questions on the agrobacterium-mediated soybean cotyledonary node systems. Soybean Sci. 27, 173–175. 10.11861/j.issn.1000-9841.2008.01.0173

[B33] Li W. Ning H. Lv W. Li W. (2008b). Optimization of the agrobacterium-mediated transformation systems of soybean cotyledonary node. Sci. Agric. Sin. 41, 971–977. 10.3864/j.issn.0578-1752.2008.04.005

[B34] Lin Y. J. Zhang Q. (2005). Optimising the tissue culture conditions for high efficiency transformation of indica rice. Plant Cell Rep. 23, 540–547. 10.1007/s00299-004-0843-6 15309499

[B35] Liu H. K. Yang C. Wei Z. M. (2004). Efficient *Agrobacterium tumefaciens*-mediated transformation of soybeans using an embryonic tip regeneration system. Planta 219, 1042–1049. 10.1007/s00425-004-1310-x 15605177

[B36] Liu Z. Shan L. Xu P. Li G. Xiuli W. (2001). Advances in genetic transformation of graminaceous crops by particle bombardment. J. Shenyang Agric. Univ. 32, 465–468. 10.1007/s00425-004-1310-x

[B37] Masekesa R. T. Gasura E. Matikiti A. Kujeke G. T. Ngadze E. Icishahayo D. (2016). Effect of BAP, NAA and GA3, either alone or in combination, on meristem culture and plantlet establishment in sweet potato (cv Brondal). Afr. J. Food Agric. Dev. 16, 10653–10669. 10.18697/ajfand.73.15875

[B38] Mccabe D. E. Swain W. F. Martinell B. J. Christou P. (1988). Stable transformation of soybean (Glycine Max) by particle acceleration. Nat. Biotechnol. 6, 923–926. 10.1038/nbt0888-923

[B39] Murashige T. Skoog F. (1962). A revised medium for rapid growth and bio assays with tobacco tissue cultures. Physiol. Plant. 15, 473–497. 10.1111/j.1399-3054.1962.tb08052.x

[B40] Nishijima T. Katsura N. Koshioka M. Yamazaki H. Mander L. N. (1997). Effects of uniconazole and GA3 on cold-induced stem elongation and flowering of *Raphanus sativus* L. Plant Growth Regul. 21, 207–214. 10.1023/A:1005806107254

[B41] Normanly J. (1997). Auxin metabolism. Physiol. Plant. 100, 431–442. 10.1111/j.1399-3054.1997.tb03047.x

[B42] Olhoft P. Somers D. (2001). L-Cysteine increases Agrobacterium -mediated T-DNA delivery into soybean cotyledonary-node cells. Plant Cell Rep. 20, 706–711. 10.1007/s002990100379

[B43] Paterson A. H. Brubaker C. L. Wendel J. F. (1993). A rapid method for extraction of cotton (*Gossypium* Spp.) genomic DNA suitable for RFLP or PCR analysis. Plant Mol. Bio. Rep. 11, 122–127. 10.1007/BF02670470

[B44] Paz M. M. Martinez J. C. Kalvig A. B. Fonger T. M. Wang K. (2006). Improved cotyledonary node method using an alternative explant derived from mature seed for efficient Agrobacterium-mediated soybean transformation. Plant Cell Rep. 25, 206–213. 10.1007/s00299-005-0048-7 16249869

[B45] Peng X. Yi Z. JIang J. Liu L. (2007). Perliminary studies on anti-browning during the tissue culture of bromeliaceae. Hunan Agric. Sci. 67–69. 10.16498/j.cnki.hnnykx

[B46] Qi W. Tinnenbroek-Capel I. E. Schaart J. G. Huang B. Cheng J. Visser R. G.. (2014). Regeneration and transformation of *Crambe abyssinica*. BMC Plant Biol. 14:235. 10.1186/s12870-014-0235-1 25195944 PMC4156612

[B47] Qiu Y. W. Gao X. J. Qi B. R. Li L. Zhen Z. (2012). Establishment of taqman real-time quantitative pcr assay for foreign gene copy numbers in transgenic soybean. J. Northeast Agric. Univ. 19, 48–52. 10.1016/S1006-8104(13)60050-1

[B48] Ren H. Nan H. Cao D. Liu X. Liu B. Kong F. (2012). Progress and perspective on soybean genetic transformation. J. Northeast Agric. Univ. 43, 6–12.

[B49] Sambrook J. F. Russell D. W. (2001). Molecular Cloning: A Laboratory Manual. New York, NY: Cold Spring Harbour Laboratory Press.

[B50] Sato S. Newell C. Kolacz K. Tredo L. Finer J. Hinchee M. (1993). Stable transformation via particle bombardment in two different soybean regeneration systems. Plant Cell Rep. 12, 408–413. 10.1007/bf00234702 24197342

[B51] Schmutz J. Cannon S. B. Schlueter J. Ma J. Mitros T. Nelson W.. (2010). Genome sequence of the palaeopolyploid soybean. Nature 463, 178–183. 10.1038/nature08670 20075913

[B52] Shou H. Palmer R. G. Wang K. (2002). Irreproducibility of the Soybean pollen-tube pathway transformation procedure. Plant Mol. Biol. Rep. 20, 325–334. 10.1007/BF02772120

[B53] Skoog F. Miller C. O. (1957). Chemical regulation of growth and organ formation in plant tissues cultured *in vitro*. Symp. Soc. Exp. Biol. 11, 118–130. 13486467

[B54] Song Z. Tian J. Fu W. Li L. Lu L. Zhou L.. (2013). Screening chinese soybean genotypes for agrobacterium-mediated genetic transformation suitability. J. Zhejiang Univ. Sci. B. 14, 289–298. 10.1631/jzus.B1200278 23549846 PMC3625525

[B55] Sun X. (2013). Establishment and Optimization of Soybean Cotyledonary-Node Genetic Transformation System of Jiyu47. Thesis, Jilin University Press.

[B56] Tang W. Newton R. J. Weidner D. A. (2007). Genetic transformation and gene silencing mediated by multiple copies of a transgene in eastern white pine. J. Exp. Bot. 58, 545–554. 10.1093/jxb/erl228 17158108

[B57] Thomas S. G. Sun T. (2004). Update on gibberellin signaling. a tale of the tall and the short. Plant Physiol. 135, 668–676. 10.1104/pp.104.040279 15208413 PMC514103

[B58] Tiwari V. Chaturvedi A. K. Mishra A. Jha B. (2015). An efficient method of agrobacterium-mediated genetic transformation and regeneration in local Indian cultivar of groundnut (*Arachis hypogaea*) using grafting. Appl. Biochem. Biotechnol. 175, 436–453. 10.1007/s12010-014-1286-3 25308617

[B59] Vodkin L. O. Rhodes P. R. Goldberg R. B. (1983). cA lectin gene insertion has the structural features of a transposable element. Cell 34, 1023–1031. 10.1016/0092-8674(83)90560-3 6313203

[B60] Wang G. Huang M. (2002). Tissue culture and plant regeneration of cerasus campanulata. J. Nanjing Univ. 26, 73–76. 10.3969/j.issn.1000-2006.2002.02.019

[B61] Wang G. L. Xu Y. N. (2008). Hypocotyl-based Agrobacterium-mediated transformation of soybean (Glycine max) and application for RNA interference. Plant Cell Rep. 27, 1177–1184. 10.1007/s00299-008-0535-8 18347801

[B62] Wang G. Sun Y. Na J. Li H. Fang H. (2006). Progress and problems of commercial production to transgenic plants in china. Sci. Agric. Sin. 39, 1328-1335. 10.3321/j.issn:0578-1752.2006.07.005

[B63] Wang G. Wang P. Lin Y. Zhang L. Wu Y. (2002). The studies of sensitivity of genotypes in soybean to lines of *Agrobacterium tumefaciens*. Heresitas 24, 297–300. 10.16288/j.yczz.2002.03.018 16126686

[B64] Wang W. Zhu C. Liu X. Chen K. Xu C. (2011). Techniques for rapid preparation of tomato leaf DNA and its application in real-time quantitative PCR-based transgene detection. Hereditas 33, 1017–1022. 10.3724/SP.J.1005.2011.01017 21951804

[B65] Wang X. Liu S. Luo Y. (2009). Research progression soybean tissue culture system and transformation. Soybean Sci. 28, 731–735. 10.11861/j.issn.1000-9841.2009.04.0731

[B66] Wei J. Chen S. Zeng L. Yang J. Liu X. Zhu X. (2011). Quantitative expression analysis of rice bacterial blight resistant candidate genes of Xa7 by real-Time fluorescent quantitative PCR. Mol. Plant Breed. 9, 9–16. 10.3969/mpb.009.000009

[B67] Weng H. Pan A. Yang L. Zhang C. Liu Z. Zhang D. (2004). Estimating number of transgene copies in transgenic rapeseed by real-time PCR assay with HMG I/Y as an endogenous reference gene. Plant Mol. Biol. Rep. 22, 289–300. 10.1007/BF02773139

[B68] Wu X. Li W. Zhang S. (2005). The research progress of transgenic soybean in china. Soybean Sci. 24, 144–149. 10.3969/j.issn.1000-9841.2005.02.013

[B69] Xiao R. G. Guo K. W. Qiao S. Yan W. Li J. A. (2007). Phytase expression in transgenic soybeans: stable transformation with a vector-less construct. Biotechnol. Lett. 29, 1781–1787. 10.1007/s10529-007-9439-x 17609861

[B70] Xu M. Li Y. Wu J. (2010). Comparative analysis of tissue culture efficiency in four varieties in limonium. Northern Hortic. 2, 157–160.

[B71] Yang A. He C. Zhang K. (2006). Improvement of Agrobacterium-mediated transformation of embryogenic calluses from maize elite inbred lines. In Vitro Cell Dev. Biol. Plant 42, 215–219. 10.1079/IVP2006768

[B72] Yang J. Xing G. Du Q. Sui L. Guo D. Niu L. (2016). Effects of different soybean genotypes on the transformation efficiency of soybean and analysis of the t-dna insertions in the soybean genome. Soybean Sci. 35, 562–567. 10.11861/j.issn.1000-9841.2016.04.0562

[B73] Yang X. (2012). Analysis of the copy number of exogenous genes in transgenic cotton using real-time quantitative PCR and the 2^−ΔΔCT^ method. Afr. J. Biotechnol. 11, 6226–6233. 10.5897/AJB11.4117

[B74] Yang X. Sui L. Li Q. Yang J. XIng G. Guo D. (2012). Recent advances in soybean transformation. Soybean Sci 31, 302–310. 10.3969/j.issn.1000-9841.2012.02.031

[B75] Yu H. Xia G. Hou B. (2005). Factors improving the efficiency of wheat transformation mediated by *Agrobacterium tumefaciens*. J. Shandong Univ. 40, 120–124. 10.3969/j.issn.1671-9352.2005.06.026

[B76] Yu Y. Liang H. Wang S. Lian Y. Wei Y. Wang T. (2010). Research progress and commercialization on transgenic soybean in china. Soybean Sci. 29, 143–150. 10.11861/j.issn.1000-9841.2010.01.0143

[B77] Yuan L. Sun H. Yang C. Shang Y. Lu X. Zhao L. (2010). Analysis of junction sequence in the transgenic maize MON88017 and the methods of qualitative PCR detection. Acta Agron. Sin. 36, 361–364. 10.3724/SP.J.1006.2010.00361

[B78] Yukika Y. Mikihiro O. Ayuko K. Atsushi H. Yuji K. Shinjiro Y. (1997). Activation of gibberellin biosynthesis and response pathways by low temperature during imbibition of *Arabidopsis thaliana* seeds. Lung Cancer 18, 367–378. 10.1105/tpc.018143 PMC341910 14729916

[B79] Zhang J. Zhao T. Zheng W. Shang L. Wang Y. Qiu L. (2016). Genetic transformation of soybean with resistance gene GmUBC2 and the identification of resistant materials. J. Agric. Univ. Hebei. 39, 106–116. 10.13320/j.cnki.jauh.2016.0043

[B80] Zhang Z. Y. Xing A. Q. Staswick P. Clemente T. E. (1999). The use of glufosinate as a selective agent in Agrobacterium-mediated transformation of soybean. Plant Cell Tiss Org 56, 37–46. 10.1023/A:1006298622969

[B81] Zhong G. (2007). Establishment of High Efficient Regenration System of Lilium and Agrobacterium-mediated Genetic Transformation. Thesis, Southwest University Press.

[B82] Zhong H. Que Q. (2009). Method for Transforming Soybean (Glycine max). United States Patent: US 20090023212.

[B83] Zhou G. Y. Weng J. Zeng Y. Huang J. Qian S. Liu G. (1983). Introduction of exogenous DNA into cotton embryos. Method Enzymol. 101, 433–481. 10.1016/0076-6879(83)01032-0 6577258

[B84] Zhou J. Wang C. Liu X. Tan Y. (2011). A study on improving regeneration rate of agrobacterium-mediated callus of mature embryo for indica rice. Chin. Agric. Sci. Bull. 27, 1–5.

