# Supplementary material for: Optimization of Agrobacterium-Mediated Transformation in Soybean
Source: Front Plant Sci. 2017 Feb 24;8:246. doi: 10.3389/fpls.2017.00246 (PMC5323423; doi:10.3389/fpls.2017.00246)
Supplement: Supplementary file 1 [file Table1.DOCX]

Supplementary Material

Optimization of *Agrobacterium*-mediated transformation in soybean

Shuxuan Li^1^, Yahui Cong^1^, Yaping Liu^1^, Tingting Wang^1^, Qin Shuai^1^, Nana Chen^1^, Junyi Gai^1^, Yan Li^1*^

^*^ Correspondence: Yan Li, [yanli1@njau.edu.cn](mailto:yanli1@njau.edu.cn)

**Table S1︱Composition of GUS staining solution.**

| Phosphate buffer (pH 7.0)  Na_2_EDTA  K_3_[Fe(CN)6]  K_4_[Fe(CN)6] | 100 mmol/L  10 mmol/L  1 mmol/L  1 mmol/L |
| --- | --- |
| TritonX-100  Methanol  ddH_2_O  X-Gluc | 0.5%(v/v)  20%(v/v)  20%(v/v)  1 mg/ml |
