# Supplementary material for: Optimization of Agrobacterium-Mediated Transformation in Soybean
Source: Front Plant Sci. 2017 Feb 24;8:246. doi: 10.3389/fpls.2017.00246 (PMC5323423; doi:10.3389/fpls.2017.00246)
Supplement: Supplementary file 2 [file Table2.DOCX]

Supplementary Material

Optimization of *Agrobacterium*-mediated transformation in soybean

Shuxuan Li^1^, Yahui Cong^1^, Yaping Liu^1^, Tingting Wang^1^, Qin Shuai^1^, Nana Chen^1^, Junyi Gai^1^, Yan Li^1*^

^*^ Correspondence: Yan Li, [yanli1@njau.edu.cn](mailto:yanli1@njau.edu.cn)

**Table S2︱Primer sequences for PCR and quantitative PCR.**

| Primer | Sequence (5’-3’) | Size (bp) |
| --- | --- | --- |
| PCR: |  |  |
| *GUS*-F  *GUS*-R  *bar*-F  *bar*-R  Quantitative PCR:  *bar*-F | ATGTTACGTCCTGTAGAAACCCC  TCATTGTTTGCCTCCCTGCTGC  AGGCTGAAGTCCAGCTGCCAGAA  TACATCGAGACAAGCACGGTCAA  ACAAGCACGGTCAACTTCC | 1812  428  175 |
| *bar*-R | ACTCGGCCGTCCAGTTCGTA |  |
| *lectin*-F | CTGGTGATCAAGTCGTCGCT | 128 |
| *lectin*-R | GTTGGCCAAATCCCAAGACG |  |
