# Supplementary material for: Optimization of Agrobacterium-Mediated Transformation in Soybean
Source: Front Plant Sci. 2017 Feb 24;8:246. doi: 10.3389/fpls.2017.00246 (PMC5323423; doi:10.3389/fpls.2017.00246)
Supplement: Supplementary file 3 [file Table3.DOCX]

Supplementary Material

Optimization of *Agrobacterium*-mediated transformation in soybean

Shuxuan Li^1^, Yahui Cong^1^, Yaping Liu^1^, Tingting Wang^1^, Qin Shuai^1^, Nana Chen^1^, Junyi Gai^1^, Yan Li^1*^

^*^ Correspondence: Yan Li, [yanli1@njau.edu.cn](mailto:yanli1@njau.edu.cn)

**Table S3︱The effect of AgNO_3_ on the rate of shoot elongation and transformation efficiency in soybean variety 88-1.**

| AgNO_3_ concentration  (mg/L) | Rate of shoot elongation (%) | Transformation efficiency（%） |
| --- | --- | --- |
| 0 | 6.35±0.01^a^ | 3.17±0.01^a^ |
| 5 | 12.78±0.03^a^ | 4.72±0.00^a^ |
| 10 | 11.00±0.01^a^ | 2.24±0.00^a^ |
| 15 | 9.98±0.00^a^ | 5.50±0.01^a^ |

The results are expressed as mean ± standard error. Forty-five explants were infected by *Agrobacterium* for each treatment and the experiments were repeated twice. The concentrations of glufosinate for selection were 5 mg/L and 3 mg/L in SIM and SEM, respectively. The numbers of elongated shoots (height ≥3cm) were recorded during SEM stage. Means with the same letter are not significantly different at 0.05 level according to Duncan’s multiple range test. Rate of shoot elongation (%) = (The number of elongated shoots / the number of infected explants) × 100%. Transformation efficiency (%) = (The number of the positive plants / the number of infected explants) × 100%.
