# Supplementary material for: Optimization of Agrobacterium-Mediated Transformation in Soybean
Source: Front Plant Sci. 2017 Feb 24;8:246. doi: 10.3389/fpls.2017.00246 (PMC5323423; doi:10.3389/fpls.2017.00246)
Supplement: Supplementary file 4 [file Image1.PDF]

Supplementary Material

Optimization of *Agrobacterium*-mediated transformation in soybean

Shuxuan Li[1], Yahui Cong[1], Yaping Liu[1], Tingting Wang[1], Qin Shuai[1], Nana Chen[1], Junyi Gai[1], Yan Li[1*]

[*] Correspondence: Yan Li, yanli1@njau.edu.cn

Supplementary Figures

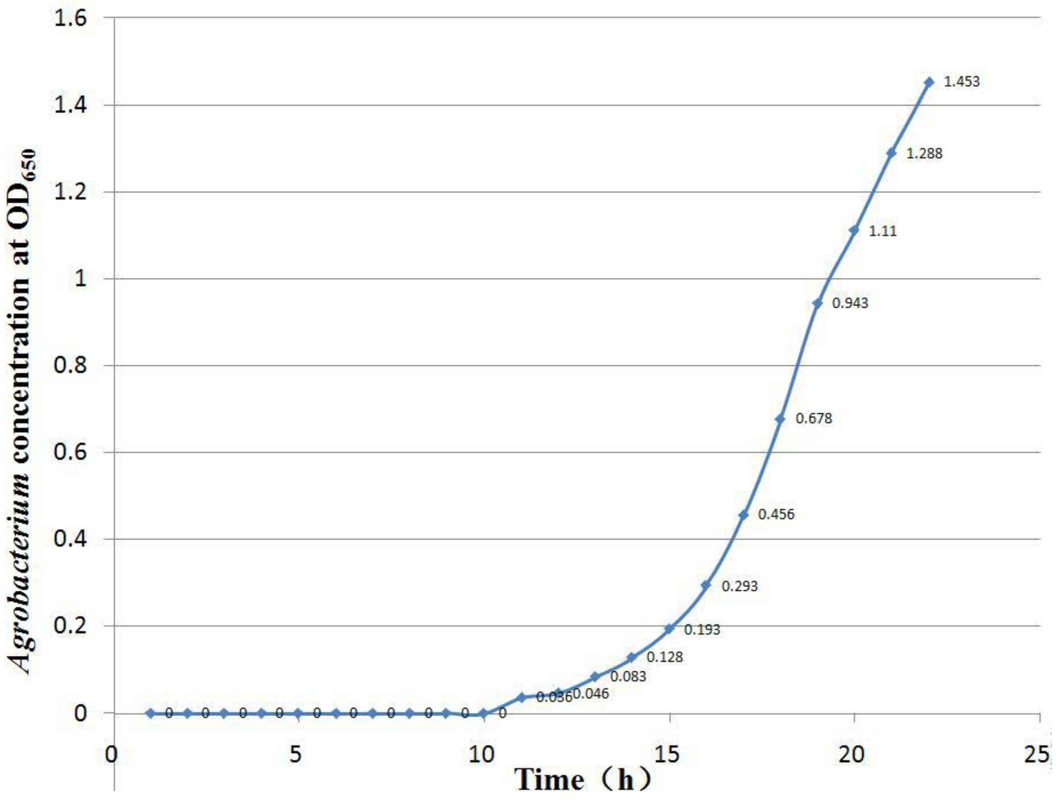

Figure S1 | The growth curve of *A. tumefaciens* strain EHA101 containing the binary plasmid pTF102.

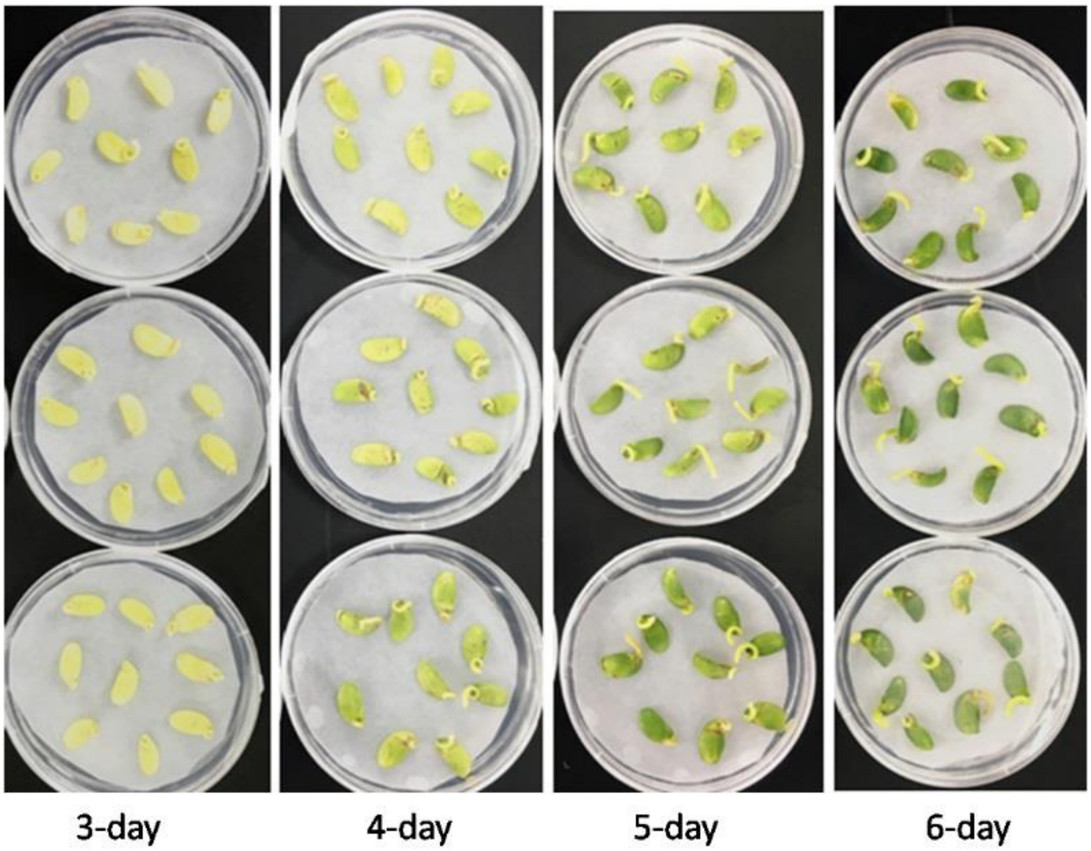

Figure S2 | The soybean cotyledonary explants after different days of co-cultivation.

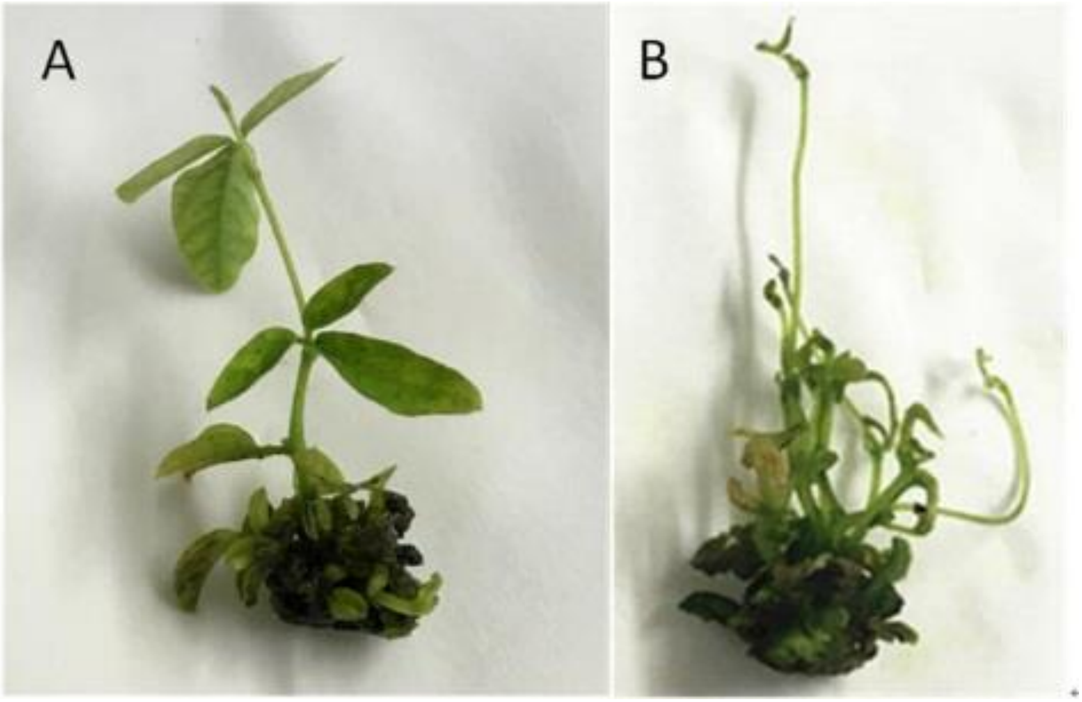

Figure S3 | The effect of $AgNO_3$ on elongated shoots of soybean variety Jack Purple. (A) The shoots were normal in the SEM without 15 mg/L $AgNO_3$. **(B)** The shoots were abnormal in the SEM with 15 mg/L $AgNO_3$.

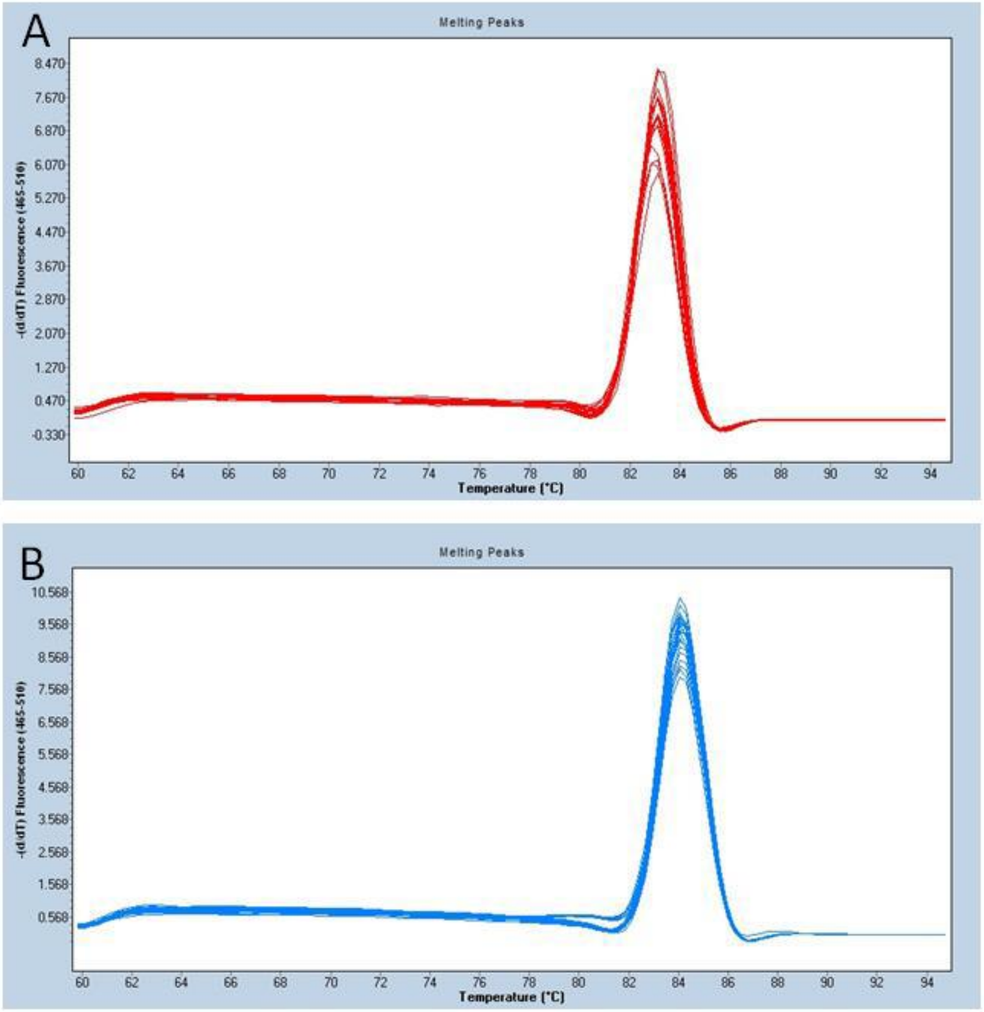

Figure S4 | The melting curves of *lectin* gene (A) and *bar* gene (B).
